# Engineering of DNA Structures Attached to Magnetic Particles for Effective Trans- and Cis-Cleavage in Cas12-Based Biosensors

**DOI:** 10.3390/ijms24054484

**Published:** 2023-02-24

**Authors:** Aleksandr V. Ivanov, Irina V. Safenkova, Sergey F. Biketov, Anatoly V. Zherdev, Boris B. Dzantiev

**Affiliations:** 1 A.N. Bach Institute of Biochemistry, Research Centre of Biotechnology of the Russian Academy of Sciences, 119071 Moscow, Russia; 2 State Research Center for Applied Microbiology & Biotechnology, 142279 Obolensk, Moscow Region, Russia

**Keywords:** CRISPR-Cas12, magnetic particles, cis-cleavage, trans-cleavage, DNA amplification

## Abstract

Sequence-specific endonuclease Cas12-based biosensors have rapidly evolved as a strong tool to detect nucleic acids. Magnetic particles (MPs) with attached DNA structures could be used as a universal platform to manipulate the DNA-cleavage activity of Cas12. Here, we propose nanostructures of trans- and cis-DNA targets immobilized on the MPs. The main advantage of the nanostructures is a rigid double-stranded DNA adaptor that distances the cleavage site from the MP surface to ensure maximum Cas12 activity. Adaptors with different lengths were compared by detecting the cleavage by fluorescence and gel electrophoresis of the released DNA fragments. The length-dependent effects for cleavage on the MPs’ surface were found both for cis- and trans-targets. For trans-DNA targets with a cleavable 15-dT tail, the results showed that the optimal range of the adaptor length was 120–300 bp. For cis-targets, we varied the length and location of the adaptor (at the PAM or spacer ends) to estimate the effect of the MP’s surface on the PAM-recognition process or R-loop formation. The sequential arrangement of an adaptor, PAM, and a spacer was preferred and required the minimum adaptor length of 3 bp. Thus, with cis-cleavage, the cleavage site can be located closer to the surface of the MPs than with trans-cleavage. The findings provide solutions for efficient Cas12-based biosensors using surface-attached DNA structures.

## 1. Introduction

CRISPR (Clustered Regularly Interspaced Short Palindromic Repeats)-Cas (CRISPR associated protein) systems are actively used as tools for bio-sensor developments [1]. Among the large Cas family, Cas12a is the most requested one for programmable and high specific recognition of DNA followed by cleavage at a certain point. In biosensors, the CRISPR-Cas comprises Cas12a and engineered guide RNA (gRNA). The gRNA contains a 5′ stem-loop and spacer region of DNA recognition [2]. The holo-enzyme recognizes a sequence complementary to the gRNA spacer and cuts each strand of target double-stranded (ds) DNA [3]. In addition, an activated Cas12a is able to facilitate the non-specific trans-cleavage of single-stranded (ss) DNA [4].

The assembled holoenzyme starts scanning dsDNA until identifying a protospacer adjacent motif (PAM) for which 5′-TTTV-3′ is the most typical sequence [2]. When the PAM is found, Cas12a unwinds 20 bp of the PAM downstream region (R-loop), and gRNA forms a complementary complex with the target strand (TS) of the R-loop [3]. Once a correct R-loop has been formed, Cas12a rapidly digests a phosphodiester bond in the non-target strand (NTS) near 3′ of the NTS spacer, which is +15–+19 nt away from PAM [3,4]. After conformation rearrangements, Cas12 digests TS at a position +22–+23 nt [2,3]. After NTS cleavage, the Cas12a active center opens to digest any ssDNA interacted with the Cas12a complex that determines trans-cleavage activity [4]. In addition, ssDNA complementary to gRNA can be recognized without PAM and cis-cleaved followed by the unspecific trans-cleavage of any ssDNA [5].

The trans-cleavage activity of Cas12a is heavily used for detecting DNA by different approaches. The first and the most widespread among them is DETECTR [4]. The number of test systems utilizing DETECTR is rising dramatically [1,6]. No less common in Cas12-based biosensors is the method called HOLMES, which was proposed almost simultaneously with DETECTR and differs mainly in the way amplicons produce before the reaction with Cas12a [7]. In Cas12-based biosensors, the recognition of DNA by Cas12-gRNA causes a trans-cleavage of the ssDNA probe being in solution or attached to the carrier surface and followed by detection of fluorescence, lateral flow assay, etc. [8]. Using an ssDNA target in a solution is a common method for Cas12-biosensors, in which the optimal ssDNA length and nucleotide composition for the most efficient trans-cleavage are known [9]. At the same time, many promising Cas12-biosensors are being designed based on different ssDNA attached to various surfaces. The parameters of biosensors with attached ssDNA are not optimized. Thus, the question about the choice of structures for surface-attached ssDNA is still open. Biosensors were constructed using a short (10–35 nt) ssDNA immobilized on gold nanoparticles (GNPs) [10], a combination of magnetic particles (MPs) and platinum nanoparticles bound with 200 nt poly-dT ssDNA [11], a combination of MPs and catalase bound with 43 nt poly-dA ssDNA [12], 29 nt ssDNA conjugated with protein (human chorionic gonadotropin) [13], the composite dsDNA–ssDNA–protein (IgG) probe attached to polystyrene microplate [14], 10–30 nt ssDNA modified by methylene blue (electrochemical tag) immobilized on the gold surface of the chip [15], and DNA hairpin (17 ds helix and 8 nt loop) with methylene blue [16]. The attachment to the surface in Cas12-biosensors is also useful for DNA cis-targets to monitor non-nucleic acid analytes [1]. Particularly, signal transduction between the recognition of an analyte and CRISPR/Cas12 amplification was realized to detect insulin [17] and prostate-specific antigen [18]. Therefore, the variety of ssDNA-attached approaches demonstrates a strong potential for the emergence of biosensors with greater sensitivity and flexible adaptation to new analytes.

Herein, we focused on MPs as universal carriers to attach to the DNA targets, which are widespread in biosensing and nanotechnology for concentration, purification, separation, directed transport, cell-transferring, etc. [19]. An obvious advantage of combining MP- and Cas12a-based approaches is the convenient manipulation with non-cleaved and released DNA. Some studies using magnetic particles and Cas of different types at the same time confirm the promise of this combination [11,12]. However, there are no comparative studies directed to the selection of optimal constructs of DNA targets immobilized on the MP surface that could be more effectively used in reactions with the cis- and trans-activity of Cas12.

In this study, we sought the universal DNA constructers conjugated with MPs for the most efficient trans- and cis-cleavage. We added a rigid dsDNA-adaptor to the DNA-target that regulated the position of the cleavage site varying adaptor length. For cis-targets, we additionally studied the location of the adaptor (at the PAM or spacer ends) in DNA–MP conjugates.

## 2. Results and Discussion

### 2.1. The Scheme of Experiments and the Choice of Adaptors for DNA Targets Conjugated with MPs for Trans- and Cis-Cleavage by Cas12a

For all experiments, we assembled a holoenzyme Cas12 with gRNA and added a cis-target, which comprised a recognition site (PAM and spacer) for the Cas12a–gRNA complex. The DNA targets for trans- and cis-cleavage activity of Cas12a were conjugated with MPs. The conjugation of DNA targets and MPs was realized via interaction between biotin at the 5′-end and streptavidin, which covalently covered the MP surface. We used model DNAs without non-canonic structures (homogenous tracts, palindrome repeats forming crucifix, extremely low or high GC content, G-quadruplex structures) (see Supplementary Information, Tables S2 and S4). The cis-targets were constructed based on the IGS fragment of *Dickeya solani* (the bacterial pathogen that causes blackleg and soft rot in potato crops). We chose two sites for cis-cleavage: site 1 (gRNA1) and site 2 (gRNA2). The gRNA1 and gRNA2 were used for comparison of the different trans-targets and cis-targets, correspondingly.

The DNA probes had different lengths of the dsDNA-adaptor (20, 40, 80, 120, 160, 300, 500, and 1000 bp; these values in nanometers are presented in Table S3, Supporting Information) and had the same ss-15-dT tail connected through a PEG linker (C3). The ssDNA could be cleaved by Cas12 at multiple sites.

**Figure 1:**
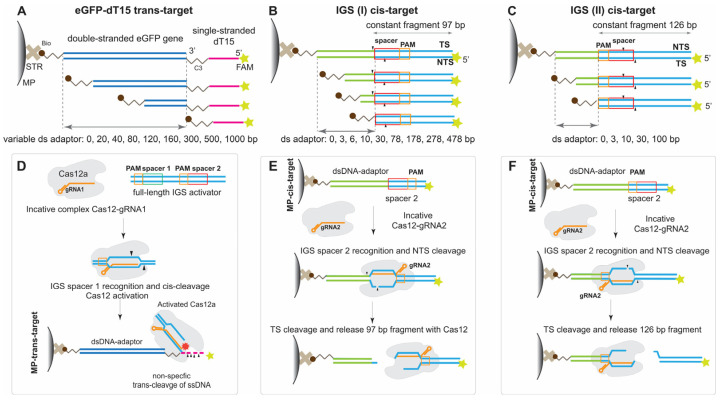
Scheme of experiments. (**A**) Biotin–dsDNA–ssDNA–FAM probe set (trans-target). (**B**) First group (IGS(I)) of cis-target constructs with a sequential arrangement of PAM, spacer, adaptor for attachment to the MP surface. (**C**) Second group (IGS(II)) of cis-target constructs with a sequential arrangement of the spacer, PAM, adaptor for attachment to the MP surface. (**D**) Trans-cleavage of the trans-target. (**E**) Cis-cleavage of the cis-target from the first group. (**F**) Cis-cleavage of cis-target from the second group. MP—magnetic particles, STR—streptavidin.

The dsDNA part of the probe was a tool for tuning the distance between the MP surface and ss trans-cleavage site. To detect the probe’s cleavage, the 5′-end of ss-15-dT was labeled with FAM as a fluorophore. The FAM/biotin-labeled ss-15-dT without any dsDNA portion was called an eGFP-0 fragment. The scheme of trans-DNA targets conjugated with MPs for trans-cleavage is presented in Figure 1A,D. The rigid dsDNA adaptors with different lengths have a different inclination for the terminal part. As found, the dsDNAs with that are longer than 150–180 bp are more flexible and strongly inclined [20,21]. However, we have considered the impact of the length of the dsDNA adaptor (see Table S3, Supplementary Information) to find the optimal distance between the ssDNA cleavage site and the MP surface.

To investigate the Cas12a activity for cis-targets at the MP surface, we proposed two groups of cis-targets (IGS constructs) differing in the sequential location of the PAM and spacer relative to the MP surface. Cas12 is an asymmetric enzyme with parts recognizing PAM and cleaving TS and NTS. Its two functional centers are arranged along a bound DNA–cis-target. Different activities could require different optimum distances. We determined optimum distances for both functional centers of Cas12 using ds-adaptors with different lengths located at the PAM or spacer ends.

For the first group of cis-targets (IGS(I)), we assumed that the sequential location was the PAM, spacer, ds-adaptor, and MP surface. In this group, a ds-adaptor with varying lengths from 0 to 478 bp (values are presented in Table S5, Supporting Information) of an IGS fragment following a 20 bp spacer was attached to the MPs through the 5′-biotin of TS. As Cas12 cleaves the cis-target in multiple sites of NTS and TS, the distance between the spacer and MP surface could affect the cleavage. The scheme of DNA targets from the first group conjugated with MPs for cis-cleavage is presented in Figure 1B,E. The distal FAM labeled dsDNA (97 bp) was the same for all cis-targets in this group. The recognition and cleavage of the MP-bound IGS construct by Cas12 led to the release of the FAM-labeled dsDNA fragment (Figure 1E).

For the second group of cis-targets (IGS(II)), we assumed that the sequential location was the spacer, PAM, the adaptor, and the MP surface. For this group, the position of PAM was close to the edge of the MP surface and could be crucial for Cas12-recognition and the following cis-cleavage. The ds-adaptor was located between the first T of PAM and 5′-biotin of NTS and had varying lengths (0, 3, 10, 30, or 100 bp of IGS fragments; these values in nanometers are presented in Table S5, Supporting Information). The distal FAM-labeled fragment had a fixed length of 126 bp. The constant fragment was released from the cis-DNA-MP conjugate upon Cas12 cleavage, while Cas12-gRNA remained bound to cis-DNA-MP (Figure 1F). The scheme of DNA targets from the second group conjugated with MPs for cis-cleavage is presented in Figure 1C,F.

The detection of trans-cleavage was performed based on the fluorescence of the released FAM. Three methods were used for cis-cleavage detection: (1) direct registration of the fluorescence of the released FAM, (2) gel electrophoresis of cleaved cis-fragments, and (3) indirect registration of the fluorescence of the cleaved FAM-dT15 BHQ1 probe when Cas12a was activated by cis-target DNA.

### 2.2. Characterization of Streptavidin–MPs and DNA–Streptavidin–MP Conjugates

**Figure 2:**
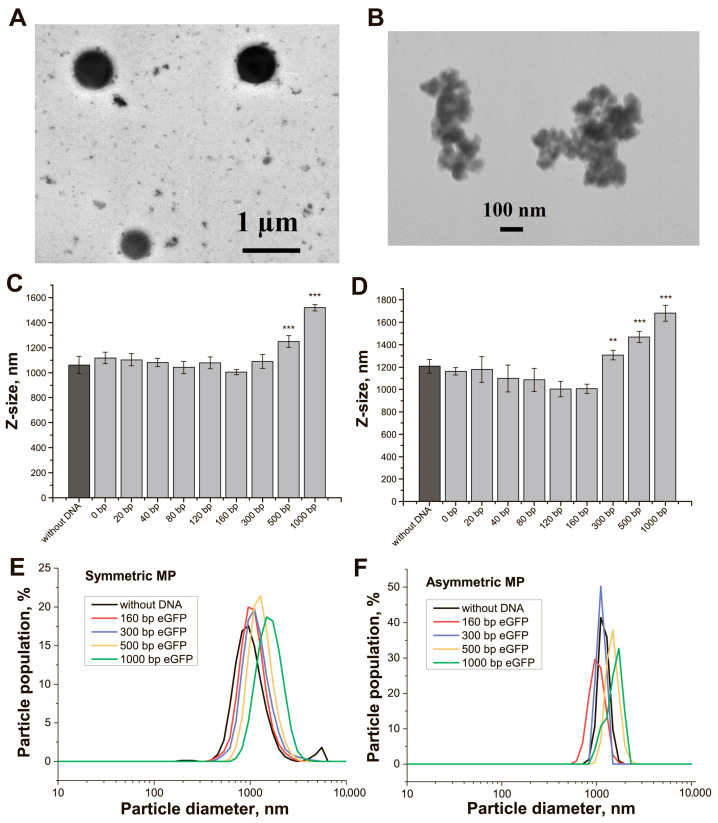
Characterization of streptavidin-MPs and their conjugates with biotinylated DNAs for trans-cleavage. TEM image of (**A**) SMPs, (**B**) AMPs. Dh values of (**C**) SMPs and their conjugates, (**D**) AMPs and their conjugates. Dh distribution for (**E**) SMPs and their conjugates, (**F**) AMPs and their conjugates. Difference from control (dark gray column): **-*p*-value < 0.05%, ***-*p*-value < 0.01%.

Two preparations of streptavidin–MPs differing in the shape and structure of the surface were used. According to TEM data, the streptavidin–MPs-1 particles had a symmetrical and spherical shape with a diameter equal to 800–1000 nm (typical particles are presented in Figure 2A); we called these particles symmetrical MPs (SMPs). The streptavidin–MPs-2 were identified as non-symmetrical particles with cavities on their surface and diameter in the range of 300–500 nm (typical particles are presented in Figure 2B); we called these particles asymmetrical MPs (AMPs). The conjugation providing the saturation of binding biotinylated DNA targets with streptavidin-MPs was already achieved at 10 min incubation (Figure S5, Supporting Information).

This optimal time was used for all conjugations. Based on both streptavidin–MPs, their conjugates were obtained with trans-targets (nine conjugates) and cis-targets (nine conjugates for the first group, five conjugates for the second group).

The hydrodynamic diameters (Dh) distribution for the streptavidin–MPs and their conjugates with DNA targets were characterized with DLS and showed homogeneity of the preparations (Figure 2C–F). The average Dh for SMPs was 1062 nm, and for AMPs, it was 1207 nm. For AMPs and their conjugates, the Dh distributions were quite narrow (Figure 2F), which corresponds to the similar hydrodynamic behavior of these particles in solution. Possibly, the irregular shape of AMPs provided an even greater influence on their behavior in solution and average translational diffusion coefficient than geometric dimensions. The fluorescence of the conjugates was estimated, and their fluorescence quenching was found (12–65% for different adaptors, Table S6, Figures S6 and S7, Supporting Information). Therefore, further evaluations were performed based on the fluorescence of unbound and released DNA targets.

For all trans-targets, we determined the percentage of bound DNA targets by MPs (loading). The loadings were in the range of 80% (eGFP-300)-96% (eGFP-0) regardless of the MP type (Table S7, Figure S8 (gray columns), Supporting Information). SMPs bound up to 54% of eGFP-500 or 34% of eGFP-1000 targets. AMPs bound to 51% of eGFP-500 or 33% of eGFP-1000 targets. Similar loadings were shown for IGS(I)-cis-targets (Table S8, Supporting Information). The effective concentration of DNA in the conjugates was estimated based on the loading that was used for further calculations.

### 2.3. Characteristics of Trans-Cleavage for DNA Trans-Targets Immobilized on the MPs

The obtained DNA–streptavidin–MP conjugates with different lengths of ds-adaptor were used as probes for trans-cleavage by Cas12a. The trans-cleavage activity of gRNA1–Cas12a complex activated by the IGS cis-target was proven with the ROX-dT15-BHQ2 probe as an internal control (Figure S10, Supporting Information, Section S7). The conjugates with a FAM-labeled ds-adaptor without ss-dT15 did not release FAM (Figure S11, Supporting Information, Section S7). Therefore, all cleavage events of the DNA–streptavidin–MPs should be a result of the cleavage of the ss-dT15 terminal part.

Before trans-cleavage experiments, we checked the effect of trans-targets: MP ratio on the trans-cleavage. The variation of the surface density of DNA (Figure S12, Supporting Information, Section S7) or the concentration of MPs (Figure S13, Supporting Information, Section S7) did not change the trans-cleavage of the conjugated targets. Therefore, we approved that the chosen concentration conditions (100 nM of trans-targets and 0.0625% of MPs) for investigation of the length-dependent regularities for trans-cleavage were in the optimal range.

**Figure 3:**
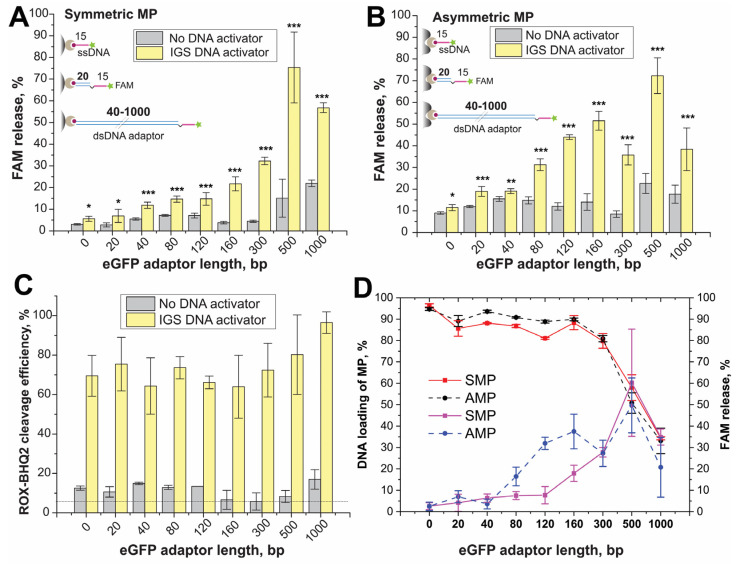
Trans-cleavage of trans-targets conjugates differing the eGFP adaptor length for (**A**) SMPs, (**B**) AMPs. Unpaired *t*-test *p*-values for corresponding raw signals are shown as asterisks: *-*p* < 0.1, **-*p* < 0.05, ***-*p* < 0.001 (**C**) Trans-cleavage of ROX–dT15–BHQ2 probe (100 nM) as internal control dash line represents the ROX–BHQ2 signal in the buffer. (**D**) Dependencies of trans-cleavage efficiency (blue and purple lines) and ds-ssDNA loading of MPs (red lines) on adaptor length. The values of negative controls were subtracted. The fluorescence of the released FAM after cleavage was normalized to an effective concentration of DNA targets in the conjugate and estimated as % of the released FAM.

For conjugates with SMPs, the efficiency of FAM released after trans-cleavage raised gradually with an increase in the ds-adaptor length (Figure 3A and Figure S14A,C, Supporting Information, Section S7). To control trans-cleavage results, we used MP conjugates and gRNA1–Cas12a complex without IGS activation (negative control) (see Figure 3).

The results for eGFP-0 and eGFP-20 did not show a significant difference that is relative to the negative control. For eGFP-40, a reliable difference was obtained between reactions with activated gRNA1-Cas12a complex and negative control. Cleavage efficiency in the range of eGFP-0 -eGFP-120 did not exceed 15%, and the differences between reactions with activated gRNA1–Cas12a complex and without were no more than two-fold. The results for eGFP-160 and eGFP-300 showed 20% and 30% of FAM release and a 5–7-fold difference with corresponding negative controls. The cleavage for eGFP-500 and eGFP-1000 demonstrated a 55–60% of FAM release that was the most effective trans-cleavage. However, negative controls of eGFP-500 and eGFP-1000 had higher FAM release (%) than others. This fluorescence for negative controls could be the result of the potential dissociation of long eGFP fragments from MPs or partial destruction of MPs under temperature, shaking and magnet separation, resulting in the release of low-affine bound DNA targets.

The cleavage of ROX-dT-BHQ2 internal control probe led to equivalent fluorescent signals (65–90%) for each reaction with activated Cas12a (Figure 3C). The absence of significant difference for trans-cleavage of ROX–dT15-BHQ2 occurring in the presence of conjugates with different trans-targets means that the type of trans-target in conjugate has no impact on trans-nuclease activity. Therefore, the efficiency of cleavage of eGFP-0–eGFP-120 immobilized on the MPs was far inferior to cleavage in a solution (see Figure 3). The longer adaptor (>120 bp) and the corresponding increment of the distance between the cleavage site and the MP surface (conjugates with eGFP-160–1000) increased the efficiency of cleavage.

For conjugates with AMPs, the efficiency of FAM releases after trans-cleavage was higher for each ds-adaptor (Figure 3B and Figure S14B,D, Supporting Information) than the corresponding one in the case of SMPs. The high fluorescence was registered even for negative controls; the FAM release % for negative controls for all ds-adaptor lengths was close to FAM release % for negative controls for SMPs with eGFP-500, -1000 (Figure 3A). This non-specific release for AMPs could be caused by the partial formation of their conjugates with DNA by less-affine adsorption forces without biotin–streptavidin interactions. For AMPs conjugates, the dependence of trans-cleavage efficiency on ds-adaptor length had a bell curve character with a maximum at 160–500 bp range (Figure 3B and Figure S14B,D).

The incomplete cleavage of the surface-attached DNAs (that the Cas12a showed) is an effect that deserves attention. The question arises as to whether such incomplete cleavage is a common feature for surface-attached DNAs that are substrates of nucleases. To clarify this, we carried out experiments with widely used DNaseI, which has endonuclease activity to ds- and ss-DNA substrates [22]. The treatment with DNAseI of ds-ssDNA–streptavidin–MP conjugates caused the release of FAM for all conjugates, but no complete cleavage was observed (Section S8, Figure S15, Supporting Information). These results showed that the effect of length-dependent cleavage for the Cas12a was determined by the specific features of Cas12a.

We propose three possible reasons for these observed length-dependent effects:

1. The close position of cleavable ssDNA to the surface could cause steric hindrance to contact with the enzyme. Local cavities could facilitate this effect.
2. The surface charge of MPs could affect the enzyme activity nearby the surface.
3. The lower trans-cleavage efficiency for long adaptors could be caused by coiling the long dsDNA. The dsDNAs with lengths longer than 150–180 bp are more flexible and more strongly inclined [21]. This dsDNA shape could mask the ssDNA-dT15 from the Cas12a-gRNA.

Summarizing the results, we concluded that the efficiency of Cas12 trans-cleavage varied depending on the distance of the ssDNA cleavage site from the MP surface (Figure 3D). The trans-targets without or with a short adaptor had a small percentage of the cleavage. For SMPs, the maximal efficiency of the cleavage was 60% for the ds-adaptor with 500 bp, while for the ds-adaptor with 1000 bp, the efficiency decreased. For AMPs, the maximal efficiency of the cleavage was 40–50% within 120–500 bp. Simultaneously, the conjugates with 500–1000 bp adaptors have significantly lower binding with both MPs than the conjugates with other proposed adaptors (Figure 3D). Therefore, the optimal lengths of the adaptors in the ds-ssDNA trans-targets were similar for conjugates based on SMPs and AMPs: 160–300 (54–102 nm, see Table S3) and 120–300 bp (40–102 nm, see Table S3), respectively.

These results can be used in Cas12-biosensors, in which an important step is to separate the cleaved parts of the ssDNA-probe in space. That will be important to realize when the ssDNA connects the carrier and the enzyme label or ligand, which are released after trans-cleavage. New possibilities in the design of the surface-attached probes will further advance the progress of Cas12-based biosensors.

### 2.4. Efficiency of Cis-Cleavage of DNA Attached to MPs through the Spacer End

The effect of the distance between cleavage sites of cis-target and conjugation point was investigated for the conjugates with different adaptor lengths (0–478 bp); the adaptor was located between a spacer and the MP surface, and the connection of the adaptor to the MP surface was ensured due to 5′-biotin of TS (IGS(I)-cis-targets). The calculated length from the MP surface and the adaptor end is presented in Table S5, Supporting Information. The exact positions of cis-cleavages vary for Cas12a in the range of +15–+19 for NTS (the sequence number of the nucleotide after PAM) and of +22–+23 for TS [2,3]. The multiple digestions (“trimming” activity) at sites near the canonical were described, mainly for TS [23]. That is an additional factor of uncertainty for cis-cleavage location. Therefore, direct attachment of the spacer (20 nt) to the MP surface without an adaptor could provide only NTS cleavage. The ds-adaptor with a length >3 bp could provide the NTS and TS cleavages. The cis-cleavage and release of cleaved IGS-FAM occurred at both NTS and TS cleavages. The corresponding PAM and spacer positions were determined for gRNA2-Cas12a. The gRNA1-Cas12a was used as a negative control because the used ds-IGS fragments had no complete gRNA1 recognition site.

**Figure 4:**
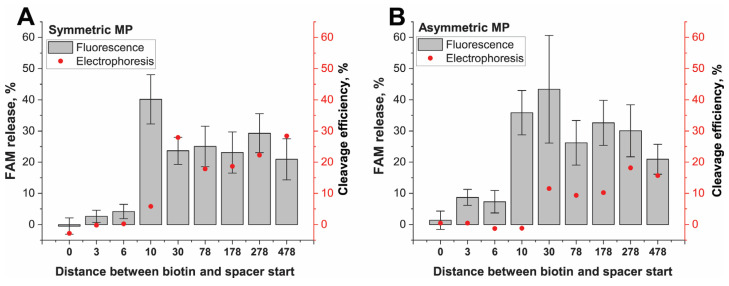
Cis-cleavage of DNA (IGS(I))–streptavidin–MP conjugates differing in the ds-adaptor length between spacer (20 bp) and the MP surface. (**A**) SMPs conjugates, (**B**) AMPs conjugates. Fluorescence values are depicted as gray bars. Efficiency values of cleavage according to gel-electrophoresis are depicted as red dots.

The cis-cleavage was performed for all proposed constructs. The results of cis-cleavage detected by the fluorescence and gel electrophoresis of released IGS-FAM fragments are presented in Figure 4 and Figure S16, Supporting Information, Section S9.

The cis-cleavage of the IGS(I)-cis-target immobilized on both types of MPs had the same features. The shortest IGS(I)-0 had no significant difference from negative controls. The IGS(I)-3 and IGS(I)-6 differed from the control, but the efficiency of the cleavage was quite small and did not exceed 5% for SMPs and 10% for AMPs (Figure 4A,B). The non-cleavage of IGS(I)-0 was associated with the impossibility of the cleavage of the TS, whereas for the IGS(I)-3 and IGS(I)-6, the possible reason was the interference of the location of the biotin–streptavidin linkage near the cleaved TS region. The IGSs with ds-adaptor lengths in the range of 10–478 bp were cleaved with an efficiency of 20–40% with high statistic deviations. Therefore, cis-cleavage efficiency did not depend on the distance between PAM and the MP surface when it exceeds 10 bp.

The direct detection of cis-cleavage by the electrophoresis of cleaved 97 bp of IGS-FAM fragments confirmed the absence of released fragments for IGS(I)-0, -3, -6 (see Figure 4 and Figure S16A–C). The FAM-containing fragment (97 bp) was very slightly released in the supernatant in the case of IGS(I)-10 (Figure 4A and Figure S16D). The released 97 bp IGS-FAM from the longer IGS fragments was more visible in gels (Figure 4 and Figure S16H,I). Gel analysis reveals similar tendencies as fluorescent measurements. However, the minimal adaptor length for effective cis-cleavage was found to be longer than the fluorescent detected one and accorded to values between 10 and 26 bp. That could be the result of the lower sensitivity of SYBR detection. In all cases, the conjugates of AMP with DNA were cleaved less effectively according to band color brightness than the conjugates of SMP with DNA. At the same time, cis-cleavage efficiency in a solution without MPs was higher than in the case of the DNA-MP conjugates. The binding of MPs with cis-targets in the region (+23)–(+26) significantly decreased the cis-cleavage efficiency, despite that both cleavages (TS and NTS) could be realized. However, cis-cleavage proceeded with similar efficiency since (+30) position binding.

We estimated the location of the DNA targets in the cleavage region of Cas12a using PDB data (6I1K) of the crystal structure of catalytically inactive FnCas12a in a complex with a crRNA and a dsDNA target [24]. According to the evidence that the cis and trans- activity of different Cas proteins are similar, we extrapolate features of a FnCas12 to the tested LbCas12a. Based on this structure, we proposed that Cas12 lobes cause steric obstacles for DNA sites located downstream of the R-loop. The lobes form a cavity that covers DNA within +26 to +29 nt from PAM (Section S10, Supporting Information). This consideration clarifies our fluorescent and electrophoretic data (Figure 4 and Figure S16) where the efficient cis-cleavage was reached when the 3′-end of PAM at NTS occurred for 30 bp or longer distances from the conjugation point. The increase in the distance between PAM 3′-end and the conjugation point up to 500 bp did not affect the cleavage efficiency in accordance with both experimental data (Figure 4) and consideration of the PDB model (Figure S18, Supporting Information).

### 2.5. Efficiency of Cis-Cleavage of DNA Attached to MPs through the PAM End

**Figure 5:**
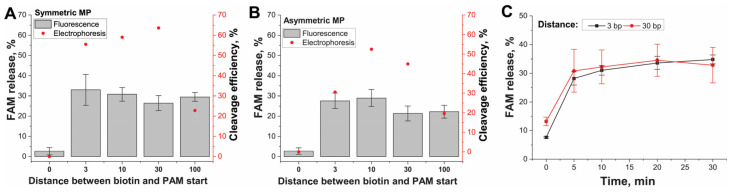
Cis-cleavage of DNA (IGS(II))–streptavidin–MP conjugates differing in the ds-adaptor length between PAM and the MP surface. (**A**) SMPs conjugates, (**B**) AMPs conjugates. Fluorescence values are depicted as gray bars. Efficiency values of cleavage according to gel electrophoresis are depicted as red dots. (**C**) Kinetic of the cis-cleavage for IGS(II)-3 and IGS(II)-30 bound to SMPs.

Finally, we investigated how close PAM can be located to the MP surface. We measured the cis-cleavage activity depending on PAM recognition at a different distance from the MP surface. The cis-cleaved IGS(II) fragment comprised two parts: (1) the same 5′-FAM-labeled distal region including an elongated spacer for gRNA2 recognition and PAM (126 bp), and (2) varied proximal region (adaptor with 0–100 bp length) following PAM and labeled with 5′-biotin attached to streptavidin–MP (Figure 1C). When Cas12 recognized and cleaved this DNA, FAM-labeled DNA was released and separated in the liquid phase. The results of cis-cleavage detected by fluorescence and gel electrophoresis of released IGS-FAM fragments are presented in Figure 5 and Figure S17, Supporting Information, Section S9.

We are using the adaptor length, since 3 bp provided cis-cleavage with 20–30% for both MPs types (Figure 5A,B) according to fluorescence. We did not find any difference in cleavage efficiency for different DNA lengths. The kinetic measurements of cis-cleavage for conjugates of SMP with 3 and 30 bp length of the proximal region demonstrated saturation after 5 min (Figure 5C). The efficiency of cleavage was similar to end-point measurements. The same results were obtained through direct gel visualization of the released DNA fragments (Figure 5A,B and Figure S17). Thus, when the PAM end of the cis-target was attached to the MP, the MP’s surface did not interfere with the Cas12a in recognizing PAM adjoining the MP surface and subsequent cis-cleavage. The conjugates with adaptors >3 bp from (-4)T of PAM demonstrated identical results of cis-cleavage. To visualize the effects of changing the adaptor length, we also considered the PDB data 6I1K for the triple complex. The visualization showed that the (-7) DNA pair position that equals 3 bp from PAM is located beyond the Cas12a globule and thus is available for interactions (Figure S18A,D, Supporting Information).

These results showed that the influence of the close location of the MP surface on PAM recognition was less than that on the cis-cleavage site close to the MP surface (Figure 4A). However, the cis-cleavages for both cis-targets groups showed the sharp change in the efficiency upon reaching an appropriate distance from the MP surface and similar constant efficiency (20–40%) for longer lengths. Summarizing all results about cis-cleavage, we concluded that the arrangement in the direction of the MP surface, PAM, and spacer was preferred for binding the cis-target to MPs. The minimal adaptor length following PAM required for this binding corresponded to 3 bp.

The orientation of cis-targets on the MP surface affects the trans-cleavage efficiency. The conjugation point caused different locations of the activated Cas12a:gRNA:DNA-cis-target complex (Figure 1E,F). We compared the trans-cleavage of an FAM-dT15-BHQ1 probe for cis-targets attached to MPs through the spacer end (IGS(I))–streptavidin–MP conjugates) and PAM end (IGS(II))–streptavidin–MP conjugates) using the minimal ds-adaptor length for both (IGS(I)-10 and IGS(II)-3) (Section S11, Supporting Information). As a result, we found that both orientations of Cas12 were able to trans-cleave the FAM–dT15–BHQ1 target (Figure S19, Supporting Information). Wherein, the trans-cleavage activity of the Cas12a-complex which was released from the MP surface (corresponded DNA (IGS(I))–streptavidin–MP conjugates) was slightly but statistically significant and exceeded the activity of the Cas12a complex that remained bound to MPs (corresponded DNA (IGS(II))–streptavidin–MP conjugates.

The revealed effectiveness of cis-cleavage of DNA attached to MPs could be useful for the design of an assay with a magnetic concentration of target dsDNA to increase assay sensitivity. Moreover, the obtained results are a reliable basis for the development of Cas12-based biosensors to detect non-DNA/RNA analytes. That can be realized when the MP–cis-target conjugate also carries a recognition receptor (for example, an antibody or an aptamer). In this case, the conjugate will be a transmitter between analyte recognition due to the receptor and signal generation due to trans cleavage to trans-cleavage by cis-activated Cas12a.

## 3. Materials and Methods

### 3.1. Materials

Information about used materials is presented in Section S1, Supporting Information.

### 3.2. Synthesis of DNA Probes for Trans-Cleavage by Cas12a (Trans-Targets)

A set of DNA probes with different lengths of the dsDNA adaptor (40, 80, 120, 160, 300, 500, and 1000 bp) and the same ss-15-dT tail connected through a PEG linker (C3) was synthesized through PCR (used primers are presented in Table S1, Figure S1, Supporting Information). The dsDNA adaptors were constructed on the base of the enhanced green fluorescent protein (eGFP) gene (the sequence data are presented in Section S2, Supporting Information) encoded in pGFP-N1 plasmid. The DNA probe with a 20 bp adaptor length was obtained by annealing. The detailed protocols of DNA probes production, purification, and characterization are presented in Section S2, Supporting Information.

### 3.3. Synthesis of DNA Targets for Activation of Cas12a (Cis-Targets)

The DNA cis-target for recognizing gRNA-Cas12a was ribosomal intergenic spacer (IGS) from *Dickeya solani* (sequence of the fragment is presented in Section S3, Supporting Information). The cis-target with 596 bp length contained fragments of full-length IGS (342 bp), and flanked regions came from the pGEM-T-IGS plasmid [25]. Two groups of cis-targets with different lengths of the IGS fragment and FAM, biotin labels at the opposite 5′-ends.

For the first group (IGS(I)), we assumed that the streptavidin–MP surface, 5′-biotinylated ds-adaptor, spacer for gRNA recognition, and PAM fragment were sequentially located. In this group, the length of the ds-adaptor was varied and comprised of 3, 6, 10, 26, 78, 178, 278, and 478 bp fragments (Section S3, Supporting Information). The IGS(I)-0 was constructed without the ds-adaptor, and the 5′-end of TS of the spacer was biotinylated for attachment to the streptavidin–MP surface. For the second group (IGS(II)), we assumed that the streptavidin–MP surface, 5′-biotinylated ds-adaptor, PAM fragment, and spacer were sequentially located. The length of the ds-adaptor was varied and comprised 3, 10, 30, and 100 bp (Section S3, Supporting Information). The IGS(II)-0 was constructed without the ds-adaptor, and the 5′-end of NTS of the PAM was biotinylated for the attachment to the streptavidin–MP surface. For both cis-target groups, detailed synthesis, purification and characterization are described in Section S3, Supporting Information, integrity and length were verified by electrophoresis (Figure S2, Supporting Information).

### 3.4. Synthesis and Verification of gRNAs

The design of gRNAs was performed by CHOPCHOP version 3 online non-profit software [26] in the area of the IGS fragment. To obtain the designed gRNAs (gRNA1, gRNA2) recognizing the IGS, in vitro transcription was performed according to Lu et al. [27] with modifications. The sequences, detailed protocol, and characterization of the obtained gRNAs are described in Section S4, Supporting Information. Length and integrity of the gRNA were verified by electrophoresis (Figure S3, Supporting Information). Activity of the gRNA was confirmed by fluorescent trans-cleavage assay (Figure S4, Supporting Information).

### 3.5. Conjugation of Trans- and Cis-Target DNAs with MPs Covered Streptavidin

Primarily, the commercial streptavidin–MPs were characterized by transmission electronic microscopy (TEM) and dynamic light scattering (DLS) (Section S5, Supporting Information). These MPs were conjugated with biotin/FAM labeled DNA targets with different concentrations (25–200 nM for trans-targets and 20 or 1 nM for cis-targets). Un-bound DNA molecules were removed during the magnetic separation of the MPs. Saturation of the conjugated MPs was estimated by measurement of FAM fluorescence in both bound and unbound DNA fractions. The detailed protocol of the conjugation is described in Section S6.1, Supporting Information). Obtained conjugates were estimated by DLS. The loading of MPs was estimated within each set of measurements of trans and cis-activity and then calculated statistically.

### 3.6. Trans-Cleavage of DNA Attached to MPs by Cas12a

A premix of gRNA-Cas12a was made according to the NEB protocol with modifications: –66 nM of gRNA1 and 66 nM of EnGene LbCas12a (NEB, Ipswich, MA, USA) in NEB2.1 buffer were mixed and incubated at 25 °C for 10 min. Then, 3.3 nM of full-length IGS cis-target was added and incubated for 30 min at 37 °C to activate the LbCas12a. Subsequently, a 500 nM ROX-dT15-BHQ2 probe (1 µL) was added as an internal control of Cas12a activity. The reaction mix with activated Cas12 (30 µL) was added to the pellet of the DNA–streptavidin–MP conjugate (2 µL, 1% MPs, 100 nM DNA). The reaction continued with shaking at 37 °C for 30 min. The cis- and trans-cleavages were stopped by the addition of EDTA up to 50 mM. The DNA–streptavidin–MP conjugate and cleave-off ss-dT-FAM were separated using the magnetic holder. The pellet with conjugate was resuspended in 25 mM Tris-HCl, pH 9.0 with 50 mM NaCl. The supernatant with cleave-off ss-dT-FAM was mixed with 70 µL of the same buffer. The fluorescence of FAM (extinction 498 nm, emission 517 nm) and ROX (excitation 578 nm, emission 604 nm) of the samples were measured using a black microplate and measurement was completed by an EnSpire multimode plate reader (PerkinElmer, Waltham, MA, USA).

### 3.7. Estimation of Cis-Cleavage of DNA Attached to MPs via Fluorescence and Gel Electrophoresis of the Released DNA Part

The direct assessment of cis-cleavage was through the fluorescence of the cleaved cis-target DNA attached to MPs after interaction with gRNA-Cas12a. The premix of gRNA-Cas12a (30 µL, 66 nM gRNA1 (or gRNA2), 66 nM EnGene LbCas12a, incubation at 25 °C for 10 min) was added to the pellet of the cis-target DNA–streptavidin–MP conjugate (20 nM cis-targets DNA, 2 µL of 1% MP suspension). Cis-cleavage was at 37 °C for 60 min upon shaking. The reaction was stopped by the addition of 40 mM EDTA. The fluorescence of FAM in the MP pellet (bound cis-target) and the supernatant (cleaved cis-target) separated by the magnet was measured as described in Section 3.6. “Trans-cleavage of DNA attached to MPs by Cas12a” with a slight modification: using 3000 flashes to count the value of fluorescence.

Additionally, the supernatants after cis-cleavage reactions were analyzed by gel electrophoresis in 2% agarose. The gels were stained by SYBR Gold and visualized by a GelDoc XR+ System (BioRad, Hercules, CA, USA) (gel scans are presented in Figures S16 and S17). The densities (D) of the lanes were estimated by TotalLab Quant (TotalLab Ltd., Great Britain, Newcastle upon Tyne, UK) and normalized by the relative density of the 100 bp lane of the ladder.

### 3.8. Statistical Data Analysis

All cis- and trans-cleavage reactions were performed at least 3 times. Mean values, standard deviations (SD), and relative SD were calculated by OriginProLab 11 (OriginLab, Northampton, MA, USA). Unpaired *t*-tests were performed for each MP conjugate (between gRNA1 and gRNA2-dependent reaction) by GraphPad (GraphPad Software, Boston, MA, USA). The full scheme of the data processing is presented in Figure S9, Supplementary Information.

## 4. Conclusions

We proposed the structures of trans- and cis-DNA targets based on the rigid dsDNA adaptor that distances the cleavage site (ss-15dT) from the MP surface. We found the dependence of cleavage efficiency on dsDNA adaptor lengths. The efficiency of trans-cleavage of the ss-15dT is directly correlated with the length of dsDNA adaptor in the case of SMPs. The use of AMPs demonstrated the bell-curve dependence of trans-cleavage efficiency on the dsDNA adaptor length.

The second found effect refers to the influence of the orientation of cis-targets on the MP surface for the recognition by Cas12a–gRNA complexes. The conjugation of the cis-DNA target in the upstream or downstream direction from a PAM affects the different stages of cis-activation, namely scanning of dsDNA or TS/NTS cleavage. The sequential arrangement of an adaptor, PAM, and a spacer was found to be preferable in providing the minimum length of the adaptor (3 bp).

The optimum of trans-targets conjugated with MPs and cleaved by Cas12a starts at 40 nm (120 bp). That is greater than the found optimum for cis-cleavage, which starts at 3.4 nm (10 bp) for the IGS(I)) cis-target and 1.02 (3 bp) for the IGS(II)) cis-target. Perhaps one reason for this difference is that the presence of the cis-target in a complex with Cas12a–gRNA makes the complex bigger and more charged. The DNA cis-target can confine the access of the active site to a trans-target located near the surface of the MP.

The revealing optimal structures of DNA immobilized on the MPs for Cas12-based biosensors will assist in designing other dispersed carriers. The found optimal structures for DNA trans-targets can be used in Cas12 biosensors combined with MPs to detect any DNA analyte that will be a cis-target or RNA analyte after its reverse transcription. Additional advantages can be obtained by changing fluorescein at the end of the trans-target to a label detectable in lower concentrations that provides greater sensitivity of the assay, for example, a quantum dot, or nanozyme others. The found optimal structures for the DNA cis-target can be effectively used in hybrid assay formats when the MP-cis-target conjugate arises as a response to some reaction (for example, the specific recognition of an analyte by antibody or aptamer) and triggers Cas12 trans-activity. Therefore, the obtained results provide more opportunities for the Cas12-based biosensors with surface-attached DNA targets.

## Data Availability

The data presented in this study are available on request from the corresponding author.

## Supplementary Materials

The supporting information can be downloaded at: https://www.mdpi.com/article/10.3390/ijms24054484/s1.
