# Supplementary material for: Engineering of DNA Structures Attached to Magnetic Particles for Effective Trans- and Cis-Cleavage in Cas12-Based Biosensors
Source: Int J Mol Sci. 2023 Feb 24;24(5):4484. doi: 10.3390/ijms24054484 (PMC10003267; doi:10.3390/ijms24054484)
Supplement: Supplementary file 1 [file ijms-24-04484-s001.zip › ijms-2154104-supplementary.pdf]

SUPPORTING INFORMATION

Engineering of DNA structures attached to magnetic particles for effective trans- and cis-cleavage in Cas12-based biosensors

Aleksandr V. Ivanov[1], Irina V. Safenkova[1,*], Sergey F. Biketov[2], Anatoly V. Zherdev[1] and Boris B. Dzantiev[1]

[1]A.N. Bach Institute of Biochemistry, Research Centre of Biotechnology of the Russian Academy of Sciences, 119071 Moscow, Russia; a.ivanov@fbras.ru (A.V.I.), safenkova@inbi.ras.ru (I.V.S.), zher-dev@inbi.ras.ru (A.V.Z.), dzantiev@inbi.ras.ru (B.B.D.)

[2]State Research Center for Applied Microbiology & Biotechnology, 142279 Obolensk, Moscow Region, Russia; biketov@obolensk.org (S.F.B.)

*Correspondence safenkova@inbi.ras.ru; Tel.: +7-495-954-2804

CONTENT

Section S1. Materials and oligonucleotides 2

Section S2. Synthesis of DNA probes for trans-cleavage by Cas12a (trans-targets) 4

Section S3. Synthesis of DNA-targets for activation of Cas12a (cis-targets) 7

Section S4. Synthesis and verification of guide RNAs 11

Section S5. Characterization of streptavidin-MPs. 13

Section S6. Optimization of the conjugation with streptavidin-MPs and DNA-targets 14

Section S7. Trans-cleavage by Cas12a 21

Section S8. Cleavage of DNA conjugated with MPs using activate-independent endonuclease (DNAseI) 25

Section S9. Detection of cis-cleavage of DNA conjugated with MPs by gel-electrophoresis 26

Section S10. Visualization of Cas12-gRNA -DNA complex 28

Section S11. Cis-cleavage of DNA conjugated with MPs 29

Section S1. Materials and oligonucleotides

Materials

Two commercial paramagnetic iron microparticle covered with streptavidin were used– Speed Bead Magnetic Streptavidin Coated Particles (Cytiva, Marlborough, MA USA) (streptavidin-MPs-1), and SiMAG-Streptavidin (Chemicell, Berlin, Germany) (streptavidin-MPs-2). Oligonucleotides with modifications (fluorescein [FAM], 5-carboxyrhodamine-X [ROX], biotin, black hole quencher-1 [BHQ1], and PEG) were synthetized by Syntol (Moscow, Russia). EnGene LbCas12a, DNAseI, T7 RNA polymerase, NTP, Monarch DNA gel extraction kit, RNA purification kit, RNAse inhibitor were purchased from NEB (Ipswich, USA). Taq polymerase, Tersus polymerase, and dNTP were obtained from Evrogen (Moscow, Russia). SYBR Gold stain were purchased from Invitrogen (Waltham, MA, USA). Centrifugal filters Amicon Ultra 3K were produced by Merk Millipore (Burlington, MA, USA). Analytical grade pure salts and organic compounds were used.

Table S1. Sequences of the primers and oligonucleotide probes for DNA constructs used in this research

**Name**	**Sequence 5'-3'**	**Purpose**
M13 F	GTTGTAAAACGACGGCCAGTG	Synthesis of cis-target with 596 bp length
M13 R	AGCGGATAACAATTTCACAC	
gRNA1 DNA F	TTTTTTTTAATACGACTCACTATAGGTAATTTCTACT AAGTGTAGATAAACGACGGCATGCGAGCGTG	Transcription of gRNA1
gRNA1 DNA R	CACGCTCGCATGCCGTCGTTTATCTACACTTAGTAG AAATTACCTATAGTGAGTCGTATTAAAAAAAA	
gRNA2 DNA F	TTTTTTAATACGACTCACTATAGGTAATTTCT ACTAAGTGTAGATGGTTAGCGTTGACCGTGCCTTT	Transcription of gRNA2
gRNA2 DNA R	AAAGGCACGGTCAACGCTAACCATCTACACTTAGT AGAAATTACCTATAGTGAGTCGTATTAAAAAA	
eGFP dT15 BHQ1 F	BHQ1-TTTTTTTTTTTTTTT-(C3 spacer)-(T-FAM)GCACCCCGACCACATGAAG	Synthesis of control eGFP fragments
eGFP-C3-FAM F	FAM-(C3 Spacer)-GCTACCCCGACCACATGAAG	
eGFP 0	FAM-TTTTTTTTTTTTTTT-Bio	Trans-target (15-dT) for conjugation with MPs
ROX-dT15-BHQ2	ROX-TTTTTTTTTTTTTTT-BHQ2	Internal control of trans-cleavage
FAM-dT15-BHQ1	FAM-TTTTTTTTTTTTTTT-BHQ1	Probe for trans-cleavage in the solution

ROX-dT	ROX-TTTTTTTTTT	ROX control
eGFP dT15 FAM F	FAM-TTTTTTTTTTTTTTT-(C3 Spacer)-GCTACCCCGACCACATGAAG	Synthesis of trans-targets (eGFP 20-1000 fragments) for conjugation with MPs
eGFP R 20 Bio	Bio-CTTCATGTGGTCGGGGTAGC	
eGFP R 40 Bio	Bio-GACTTGAAGAAGTCGTGCTG	
eGFP R 80 Bio	Bio-GAAGATGGTGCGCTCCTGGA	
eGFP R 120 Bio	Bio-TCACCTCGGCGCGGGTCTTG	
eGFP R 160 Bio	Bio-TTCAGCTCGATGCGGTTCAC	
eGFP R 300 Bio	Bio-TCGATGTTGTGGCGGATCTTG	
eGFP R 500 Bio	Bio-TTACTTGTACAGCTCGTCCATGCCG	
eGFP R 1000 Bio	Bio-AAACTTGATTAGGGTGATGGTTCAC	
IGS FAM F	FAM-GTTTCAAATTATTCAGCTTGTTCCGG	Synthesis of cis-targets (IGS 0-478 fragments) with sequential arrangement of PAM, spacer, adaptor for attachment to the MP surface s
IGS R 0 Bio	Bio-AGGCACGGTCAACGCTAAC	
IGS R 3 Bio	Bio-GAAAGGCACGGTCAACGCT	
IGS R 6 Bio	Bio-GGTGAAAGGCACGGTCAAC	
IGS R 10 Bio	Bio-GGTGGGTGAAAGGCACGG	
IGS R 26 Bio	Bio-CGCCAATATGACTGACGGTG	
IGS R 78 Bio	Bio-AGGACACCGCCCTTTCACGG	
IGS R 178 Bio	Bio-TTGAAGTGCCTGCGTGGTGT	
IGS R 278 Bio	Bio-CCAAGCTATTTAGGTGACACTA	
IGS R 478 Bio	Bio-GCGTTGGCCGATTCATTAATGC	
IGS PAM F-100 Bio	Bio- GGCACACAAACTCACGCTC	Synthesis of cis-targets (IGS 0-30 fragments) with sequential arrangement of the spacer, PAM, adaptor for attachment to the MP surface
IGS PAM F-30 Bio	Bio-ATAACATAAACCTGACTATCTCTAATCAG	
IGS PAM F-10 Bio	Bio-TCTAATCAGTTTTAGGTTAGCGTTGAC	
IGS PAM F-3 Bio	Bio-AGTTTTAGGTTAGCGTTGACC	
IGS PAM F 0	Bio-TTTAGGTTAGCGTTGACCGTGC	
IGS PAM R FAM	FAM- AGTGATGTCCCCTTCGTCT	

* Bio – biotin, FAM – fluorescein, ROX – 5-carboxyrhodamine-X, T-FAM – internal dT labeled by FAM, C3 spacer – propandiol

Section S2. Synthesis of DNA probes for trans-cleavage by Cas12a (trans-targets)

2.1. Sequences

Sequences of eGFP fragments correspond to double-strand fragments. To simplify, coding strands (5'->3') are shown only. Fragment eGFP 1000 comprises all eGFP fragments with smaller lengths, so their positions along the eGFP 1000 are color marked. Yellow – eGFP 20, light green – eGFP 40, cyan – eGFP 80, pink – eGFP 120, red – eGFP 160, olive – eGFP 300, light grey – eGFP 500, grey – eGFP 1000.

Sequence of eGFP 1000 fragment (start at G221 of eGFP gene):

GCTACCCCGACCACATGAAGCAGCACGACTTCTTCAAGTCCGCCATGCCCGAAGGCTACGTCCAGGAGC
GCACCATCTTCTTCAAGGACGACGGCAACTACAAGACCCGCGCCGAGGTGAAGTTCGAGGGCGACACC
CTGGTGAACCGCATCGAGCTGAAGGGCATCGACTTCAAGGAGGACGGCAACATCCTGGGGCACAAGCT
GGAGTACAACTACAACAGCCACAACGTCTATATCATGGCCGACAAGCAGAAGAACGGCATCAAGGTGA
ACTTCAAGATCCGCCACAACATCGAGGACGGCAGCGTGCAGCTCGCCGACCACTACCAGCAGAACACCC
CCATCGGCGACGGCCCCGTGCTGCTGCCCGACAACCACTACCTGAGCACCCAGTCCGCCCTGAGCAAAG
ACCCCAACGAGAAGCGCGATCACATGGTCCTGCTGGAGTTCGTGACCGCCGCCGGGATCACTCTCGGCA
TGGACGAGCTGTACAAGTAA)AGCGGCCGCGACTCTAGATCATAATCAGCCATACCACATTTGTAGAGG
TTTTACTTGCTTTAAAAAACCTCCCACACCTCCCCCTGAACCTGAAACATAAAATGAATGCAATTGTTGTT
GTTAACTTGTTTATTGCAGCTTATAATGGTTACAAATAAAGCAATAGCATCACAAATTTCACAAATAAAG
CATTTTTTTCACTGCATTCTAGTTGTGGTTTGTCCAAACTCATCAATGTATCTTAAGGCGTAAATTGTAAG
CGTTAATATTTTGTTAAAATTCGCGTTAAATTTTTGTTAAATCAGCTCATTTTTTAACCAATAGGCCGAAAT
CGGCAAAATCCCTTATAAATCAAAAGAATAGACCGAGATAGGGTTGAGTGTTGTTCCAGTTTGGAACAA
GAGTCCACTATTAAAGAACGTGGACTCCAACGTCAAAGGGCGAAAAACCGTCTATCAGGGCGATGGCC
CACTACGTGAACCATCACCCTAATCAAGTTT

Table S2. Characterization of DNA structures for trans-cleavage

	Feature					
	GC%*	Tandem repeats (>=4)	Poly(GC)$_2$ (higher Z DNA fold)	Crucifix palindrome**	poly site (>=6)	G-quadruplex***
eGFP-20	60	0	No	low probability: ΔG >0 (5.14, 14.15)	0	low probability: 1 potential G-quadruplex with G-score 8 -13.
eGFP-40	54	0	No		0	
eGFP-80	59	0	No		0	
eGFP-120	59	0	No		0	
eGFP-160	59	0	No		0	
eGFP-300	55	0	No		0	
eGFP-500	59	0	No		0	
eGFP-1000	47	1 (aatg)$_2$	No	low probability: ΔG >0 (7.65, 11.60)	3 (A/T)	

*https://www.biologicscorp.com/tools/GCContent/
**http://palindromes.ibp.cz/ settings: size (5-30), spacer (4-10), mismatches (0,0). The indicated ΔG values accord to energy of potential folding of palindrome(s) from linear to crucifix form
*** https://bioinformatics.ramapo.edu/QGRS/index.php, maximal G-score of the algorithm is 105

Table S3. Approximate parameters of DNA trans-targets conjugated with MPs.

Trans-target	Dimensions, nm*				
	Biotin linker	dsDNA	C3 linker	ssDNA	Total length of trans-target
eGFP-0	1.82	0	0.6	10.14	12.6
eGFP-20	1.82	6.8	0.6	10.14	19.4
eGFP-40	1.82	13.6	0.6	10.14	26.2
eGFP-80	1.82	27.2	0.6	10.14	39.8
eGFP-120	1.82	40.8	0.6	10.14	53.4
eGFP-160	1.82	54.4	0.6	10.14	67.0
eGFP-300	1.82	102	0.6	10.14	114.6
eGFP-500	1.82	170	0.6	10.14	182.6
eGFP-1000	1.82	340	0.6	10.14	352.6

*Maximal lengths of C3 spacer and Biotin linker were calculated by using of bonds length: C-O = 0.143 nm, C-C = 0.154 nm, C-N before peptide bond = 0.147, C-N in peptide bond = 0.132 nm, C-C after peptide bond = 0.153. Maximal length of ds and ssDNA was calculated by multiplication of bp/nt numbers on its length. 1 bp in dsDNA = 0.34 nm, 1 nt in ssDNA = 0.676 nm.

2.2. Methods

The DNA-probes with 20 bp length of the ds fragment and the same ss-15-dT were obtained by annealing at 80 °C during 2 min of 10 µM complementary oligonucleotides – eGFP-R-20-Bio with either eGFP-C3-dT15-FAM F or eGFP-FAM-dT15-BHQ1 F (Table S1, Supporting Information), after which the mix was gradually cooled until reaching 20 °C. All target DNAs were labeled with biotin at one 5′-end and carrying FAM at opposite 5′-end of 15-dT.

Synthesis of DNA-probes with ds DNA fragment 40 – 1000 bp was performed by PCR with following conditions. The reaction mix (300 µL) contained 200 nM of dNTPs, 500 nM labeled primers, 400 ng of pGFP-N1 plasmid, 25 units of Taq polymerase, and commercial buffer (Evrogen). Seven biotinylated primers providing different length of amplicons (40 – 1000 bp) were used as reverse (R), wherein the same forward (F) eGFP-C3-dT15-FAM primer was used (see **Table S1**). Primers eGFP-C3-FAM F and eGFP-R-160-Bio primers (**Table S1**) were used to synthesize negative control of DNA target without ss-dT tail. Primers eGFP-dT15-BHQ1-F and eGFP-R-160-Bio (**Table S1**) were used to synthesize DNA target for trans-cleavage control without magnetic particles.

The PCR was performed within 40 cycles using the BioRad T100 Thermal Cycler (Biorad, Hercules, CA, USA). Each cycle comprises 30-sec denaturation at 95°C, then 30-sec primer annealing at 65°C, and 60-sec elongation at 72°C. The products of several PCR reactions were

combined into one mix to a final volume of 1 mL and purified. The mix was concentrated by Amicon Ultra 3K (Merk, Burlington, MA, USA) then purified by gel electrophoresis in 2% agarose in 20 mM Tris-acetate buffer with 0.2 mM EDTA, pH 8.3 (TAE). The gel pieces with bands corresponding target length were excised and DNAs were extracted by Monarch DNA gel extraction kit. The concentration (ng/µL) of the target DNA was estimated by measuring in triplicates with NanoDrop ND-2000 (Thermo Scientific, Waltham, MA, USA) and then converted to molar concentrations using the molar mass of the obtained DNA constructs.

The obtained target DNAs were verified by gel electrophoresis in 2% agarose in TAE with staining by ethidium bromide (4 µg/mL) and registration of fluorescence by GelDoc XR+ System (BioRad, Hercules, CA, USA) (**Figure S1**).

2.3. Results

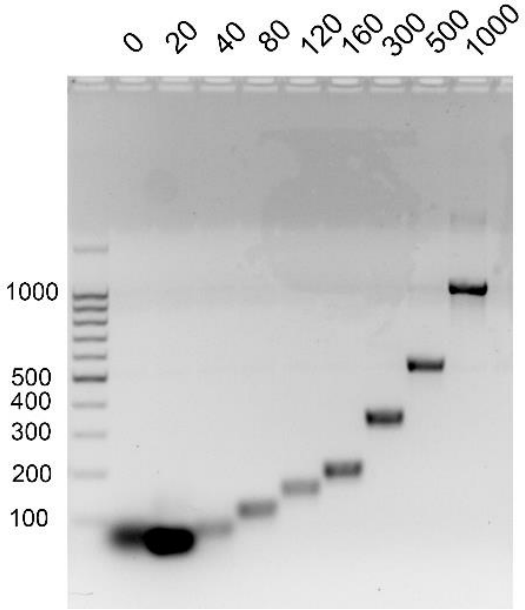

Figure S1. Verification of synthetized and purified trans-targets by electrophoresis in 2% TAE-agarose. Staining with ethidium bromide.

Section S3. Synthesis of DNA-targets for activation of Cas12a (cis-targets)

3.1. Sequences

Sequences of IGS cis-targets correspond to double-strand fragments. To simplify, coding strands (5'->3') are shown only. IGS region of *D.solani* is colored light grey. Double underline denotes PAM motive (5'-TTTA-3'). Thin underline denotes gRNA1 recognition site (along complemetary strand in this cis-target DNA). Thick underline denotes gRNA2 recognition site.

Cis-target with 596 bp length was target sequnce (TS) for gRNA1, and non-target sequnce (NTS) for gRNA2.

5'-
ACGTTGTAAAACGACGGCCAGTGAATTGTAATACGACTCACTATAGGGCGAATTGGGCCCGACGTCGCA
TGCTCCCGGCCGCCATGGCGGCCGCGGGAATTCGATGAAAGTGTCTCGGGATGCGGGTATATTGAGAG
ACTCGACCGGCACACAAACTCACGCTCGCATGCCGTCGTTTCAAATTATTCAGCTTGTTCCGGATTGTTA
AAGAGCAGATAACATAAACCTGACTATCTCTAATCAGTTTTAGGTTAGCGTTGACCGTGCCTTTCACCCA
CCGTCAGTCATATTGGCGTCCCCTAGGGGATTCGAACCCCTGTTACCGCCGTGAAAGGGCGGTGTCCTG
GGCCTCTAGACGAAGGGGACATCACTTGTCAGCTTCGCAAGACGCTTTTGACTCTTTCTTATCATCAGAC
AATCTGTGTGGACACCACGCAGGCACTTCAAATCACTAGTGAATTCGCGGCCGCCTGCAGGTCGACCAT
ATGGGAGAGCTCCCAACGCGTTGGATGCATAGCTTGAGTATTCTATAGTGTCACCTAAATAGCTTGGCG
TAATCATGGTCATAGCTGTTTCCTGTGTGAAATTGTTATCCGCT-3'

Fragment IGS 500 comprises rest of IGS fragments, so their 3' positions along the IGS are marked (compimentary to IGS R primers). Thick underline – IGS 0 (length of cis-target without adaptor – 95 bp), yellow – IGS 3 (length of cis-target with adaptor – 98 bp), *itallic font* – IGS 6 (length – 100 bp), **bold font** – IGS 10 (length – 105 bp), light green – IGS 26 (length – 121 bp), cyan – IGS 78 (length – 171 bp), red – IGS 178 (length – 273 bp), olive – IGS 278 (length – 373 bp), grey – IGS 478 (length – 573 bp). ↑ shows cleavage location of non-target (FAM-labeled) strand, ↓ shows cleavage location of target (biotin-labeled) strand. The cleavage sites are shown according to data from the LbCas12a manufacturer (NEB, USA).

IGS 500:

5'FAM-
GTTTCAAATTATTCAGCTTGTTCCGGATTGTTAAAGAGCAGATAACATAAACCTGACTATCTCTA
ATCAGTTTTAG*GTTAGCGTTGA****CCGTGCC↑TTTC↓ACC*****CACC**GTCAGTCATATTGGCGTCCCCT
AGGGGATTCGAACCCCTGTTACCGCCGTGAAAGGGCGGTGTCCTGGGCCTCTAGACGAAGGGG
ACATCACTTGTCAGCTTCGCAAGACGCTTTTGACTCTTTCTTATCATCAGACAATCTGTGTGGACA
CCACGCAGGCACTTCAAATCACTAGTGAATTCGCGGCCGCCTGCAGGTCGACCATATGGGAGA
GCTCCCAACGCGTTGGATGCATAGCTTGAGTATTCTATAGTGTCACCTAAATAGCTTGGCGTAAT
CATGGTCATAGCTGTTTCCTGTGTGAAATTGTTATCCGCTCACAATTCCACACAACATACGAGCC
GGAAGCATAAAGTGTAAAGCCTGGGGTGCCTAATGAGTGAGCTAACTCACATTAATTGCGTTGC
GCTCACTGCCCGCTTTCCAGTCGGGAAACCTGTCGTGCCAGCTGCATTAATGAATCGGCCAACG
C

Fragment IGS PAM 100 comprises rest of IGS PAM, so their 5' positions along the IGS PAM are marked by colors: yellow – IGS PAM 100, green – IGS PAM 30, cyan – IGS PAM 10, magenta – IGS

PAM 3, blue- IGS PAM 0. Double underline denotes PAM motive (5'-TTTA-3'). Thin underline denotes gRNA1 recognition site (along complemetary strand in this activator DNA). Thick underline denotes gRNA2 recognition site.

IGS PAM 100:

5'Bio-
GGCACACAAACTCACGCTCGCATGCCGTCGTTTCAAATTATTCAGCTTGTTCCGGATTGTTAAAGAGCAG
ATAACATAAACCTGACTATCTCTAATCAGTTTTAGGTTAGCGTTGACCGTGCCTTTCACCCACCGTCAGTC
ATATTGGCGTCCCCTAGGGGATTCGAACCCCTGTTACCGCCGTGAAAGGGCGGTGTCCTGGGCCTCTAG
ACGAAGGGGACATCACT

Table S4. Characterization of DNA structures for cis-cleavage

	Feature					
	GC %*	Tandem repeats (>=4)	Poly(GC)$_2$ (higher Z DNA fold)	Crucifix palindrome**	poly site (>=6)	G-quadruplex***
IGS(I)-0	37	0	No	low probability: ΔG >0 (12.18)	0	No predicted G-quadruplex
IGS(I)-3	37	0	No		0	
IGS(I)-6	39	0	No		0	
IGS(I)-10	39	1 (caac)$_2$	No		0	
IGS(I)-26	41	1(gtca)$_2$	No	low probability: ΔG >0 (10.38, 11.19)	0	
IGS(I)-78	47		No		0	
IGS(I)-178	48	1 (tctt)$_2$	No		0	
IGS(I)-278	47		No	low probability: ΔG >0 (6.52)	0	low probability: 1 potential G-quadruplex with G-score 8 -11.
IGS(I)-478	48	1(tgag)$_2$	No	low probability: ΔG >0 (6.54, 13.45)	0	
IGS(II)-0	47	1(cacc)$_2$, 1(gtca)$_2$	No	low probability: ΔG >0 (10.38, 11.19)	0	
IGS(II)-3	47		No		0	
IGS(II)-10	47		No		0	
IGS(II)-30	52		No		0	
IGS(II)-100	50		No	low probability: ΔG >0 (12.18)	0	

*https://www.biologicscorp.com/tools/GCContent/
**http://palindromes.ibp.cz/ settings: size (5-30), spacer (4-10), mismatches (0,0). The indicated ΔG values accord to energy of potential folding of palindrome(s) from linear to crucifix form
*** https://bioinformatics.ramapo.edu/QGRS/index.php, maximal G-score of the algorithm is 105

Table S5. Approximate parameters of DNA cis-targets conjugated with MPs.

Cis-target	Dimensions, nm*		
	Biotin linker	dsDNA	Total
IGS(I)-0	1.82	0	1.82
IGS(I)-3	1.82	1.02	2.84
IGS(I)-6	1.82	2.04	3.86
IGS(I)-10	1.82	3.4	5.22

IGS(I)-26	1.82	10.2	12.02
IGS(I)-78	1.82	26.52	28.34
IGS(I)-178	1.82	60.52	62.34
IGS(I)-278	1.82	94.52	96.34
IGS(I)-478	1.82	162.52	164.34
IGS(II)-0	1.82	0	1.82
IGS(II)-3	1.82	1.02	2.84
IGS(II)-10	1.82	3.4	5.22
IGS(II)-30	1.82	10.2	12.02
IGS(II)-100	1.82	34	35.82

*Maximal lengths of C3 spacer and Biotin linker were calculated by using of bonds length: C-O = 0.143 nm, C-C = 0.154 nm, C-N before peptide bond = 0.147, C-N in peptide bond = 0.132 nm, C-C after peptide bond = 0.153. Maximal length of ds and ssDNA was calculated by multiplication of bp/nt numbers on its length. 1 bp in dsDNA = 0.34 nm, 1 nt in ssDNA = 0.676 nm.

3.2. Methods

The reaction mix (300 µL) contained 200 nM dNTPs, 200 nM M13 forward and M13 reverse primers (**Table S1**), 450 ng of pGEM-IGS plasmid, 30 units of Tersus polymerase, and Tersus buffer (Evrogen). PCR was performed within 40 cycles using the BioRad T100 Thermal Cycler (BioRad, Hercules, CA USA). Each cycle comprises 30-sec denaturation at 95°C, then 30-sec primer annealing at 55°C, and 60-sec elongation at 72°C. The target product was purified by gel electrophoresis in 1.5% (or 2% for shortened cis-targets) agarose in TAE then extracted by Monarch DNA gel extraction kit. The IGS contained fragments of full-length IGS (342 bp), and flanked regions came from the plasmid. As a result, the total length of the IGS construct was 596 bp. The concentration of the DNA was estimated using NanoDrop ND-2000 (Thermo Scientific, Waltham, MA USA). The IGS fragment was verified by sequencing (Syntol, Moscow, Russia).

The cis-target (IGS(I)) for direct detection of cis-cleavage was fragment of the IGS with deletion from 1 to 69 bp (**see Supplementary Information, Section 3.1**). The fragments containing the recognition site for Cas12a-gRNA different length were labeled FAM and biotin at the opposite 5′-ends. The fragments were synthetized by PCR using forward FAM-labeled primer IGS-FAM and set of nine reverse biotin-labeled primers IGS R (0-478) Bio (see **Table S1**) amplified by PCR from pGEM-T-IGS plasmid. The reaction mix contained commercial Tersus buffer (Evrogen), 200 nM dNTPs, 500 nM forward reverse primers, 200 ng of pGEM-T-IGS plasmid, 30 units of Tersus polymerase. Total volume of PCR was 1 mL. PCR was performed within 40 cycles. Each cycle comprises 30 sec denaturation at 95°C, then 30 sec primer annealing at 60°C and 60 sec elongation at 72°C. Mix was concentrated by Amicon Ultra then purified by electrophoresis in 2% TAE-agarose gel. Target products were excised and extracted from the gel by Monarch DNA gel extraction kit. Concentration of the fragments were estimated optically (see above). Obtained

fragments were analyzed by electrophoresis in 2% TAE-agarose with staining by ethidium bromide and verified by GelDoc XR+ System (BioRad, Hercules, CA, USA) (**Figure S2A**).

Synthesis of cis-target DNA (IGS(II)) with five different lengths between PAM of gRNA2 and biotinylated terminal of the DNA was performed as described above with some modifications. The fragments were synthetized by PCR using reverse FAM-labeled primer IGS PAM R FAM and set of five forward biotin-labeled primers IGS PAM F (-100 – 0) Bio (see **Table S1**) amplified by PCR from pGEM-T-IGS plasmid. The composition of reaction mixture, reaction condition and purification were used the same as for synthesis of cis-target DNA (IGS(I)). The obtained constructs were verified by electrophoresis in 2% agarose-TAE (**Figure S2B**).

3.3. Results

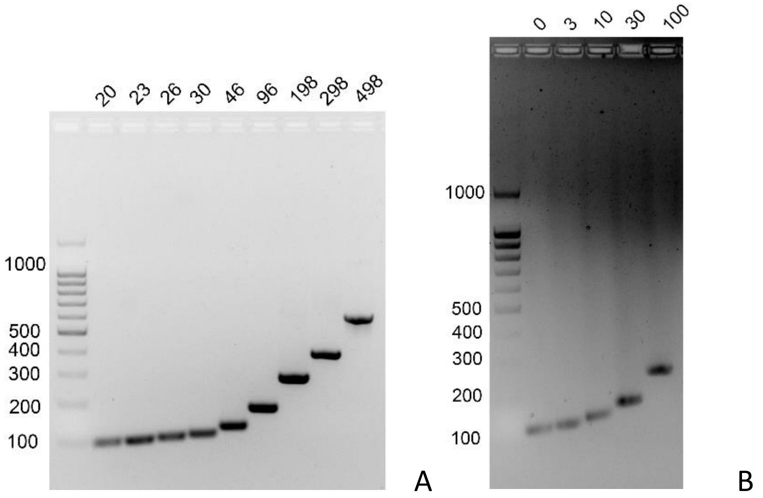

Figure S2. Verification of synthetized and purified cis-targets by electrophoresis in 2% TAE-agarose: cis-targets from IGS(I)-group recognized gRNA2 (A), cis-targets from IGS(II)-group recognized gRNA2 (B). Staining with ethidium bromide.

Section S4. Synthesis and verification of guide RNAs

4.1. Sequences

gRNA1:

5′-GGUAAUUUCUACUAAGUGUAGAU<u>AAACGACGGCAUGCGAGCGUG</u> – 3′

gRNA2:

5′- GGUAAUUUCUACUAAGUGUAGAU<u>GGUUAGCGUUGACCGUGCCUUU</u> – 3′

Site of recognition is denoted by <u>thick underline</u>

4.2. Methods

Complementary oligonucleotides (gRNA1 DNA F and gRNA1 DNA R, gRNA2 DNA F and gRNA2 DNA R, see **Table S1**) containing T7 promoter followed by 20 bp gRNA gene were denatured at 95 °C then annealed upon gradually decreasing temperature to obtain dsDNA template for in vitro transcription. The reaction mix (100 µL) contained 40 mM Tris-HCl, pH 8.0, 20 mM $MgCl_2$, 2 mM spermidine, 10 mM DTT, 1.25 mM of each NTP, 0.5 u/µL of RNAse inhibitor, 2.5 u/µL of T7 RNA polymerase, and 2 µM of dsDNA template. The reaction was performed at 37°C for 4 h. To remove residuals of the dsDNA template, 10 µL of DNAseI buffer and 3 U of DNAseI were added and incubated for 30 min at 37°C. The obtained RNAs were purified by RNA cleanup kit (NEB) according to the manufacturer’s protocol. Then DNAse treatment and RNA purification were repeated. The concentrations of the gRNAs were estimated using NanoDrop ND-2000 (USA). The integrity of the gRNAs was checked by gel electrophoresis in 15% polyacrylamide with 7M urea in 20 mM Tris-borate buffer with 0.2 mM EDTA (**Figure S3**). The functionality of the gRNAs was estimated by the trans-cleavage reaction performed according to the Cas12a manufacturer’s protocol (NEB, Ipswitch, MA, USA). Briefly, NEB2.1 buffer with 33 nM of gRNA, 33 nM of EnGene LbCas12a was incubated for 10 min at 25°C, then 1.75 µM cis-target (IGS dsDNA with 596 bp length containing recognition sites for both gRNAs) and 500 nM FAM-dT15-BHQ1 probe were added and incubated for 40 min at 37°C. The total volume was 30 µL. During the incubation, simultaneous detection of fluorescence (extinction 498 nm, emission 517 nm) of the cleaved probe was performed by LightCycler96 (Roche, Basel, Switzerland) (**Figure S4**).

4.3. Results

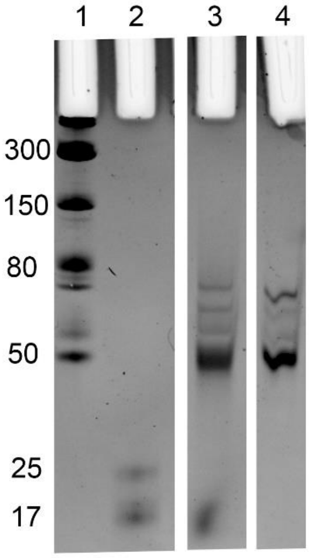

Figure S3. Electrophoresis of *in vitro* transcribed, DNAse treated and purified gRNA1 and gRNA2. 120 ng of each gRNA were loaded on 15% PAAG with7M urea. Staining by ethidium bromide. 1. ssRNA ladder low range (NEB #N0364), 2. miRNA marker (NEB #N2102S), 3. gRNA1, 4. gRNA2

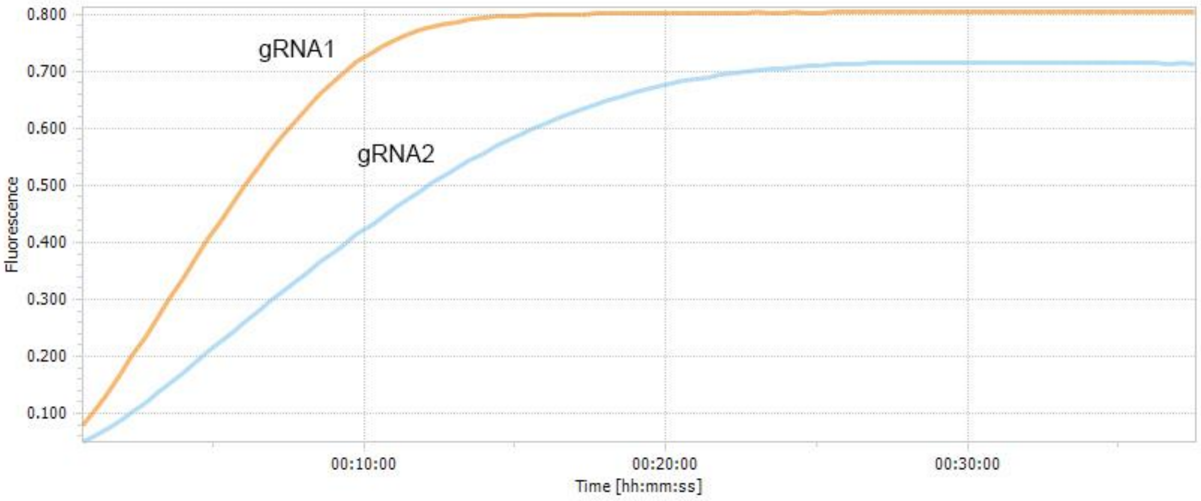

Figure S4. Fluorescence curves of FAM-dT15-BHQ1 in trans-cleavage assay of Cas12a with gRNA1 and gRNA2

The CHOPCHOP predicted the effectivity of the recognition and cis-cleavage for site 2 more than twice as compared with site 1. However, experimental data for trans-cleavage of FAM-dT15-BHQ1 showed higher activity for the case of gRNA1 (see Figure S4).

Section S5. Characterization of streptavidin-MPs.

5.1. Methods

Characterization of streptavidin-MPs by transmission electron microscopy (TEM)

The streptavidin-MPs were characterized by JEM CX-100 transmission electron microscope (Jeol, Tokyo, Japan) at an accelerating voltage of 80 kV. The glow-discharged copper grids 300 mesh (Ted Pella, USA) covered with formvar layer were used as a support for TEM. The 2 µL of each of the streptavidin-MP suspensions were diluted in 30 µL of deionized (Simplicity UV, Millipore, Burlington, MA, USA) mQ water, then 10 µL were applied on the grid and incubated for 20 min. Excess of MPs was removed by mQ rinsing.

Characterization of streptavidin-MPs and DNA – streptavidin-MP conjugates by dynamic light scattering (DLS)

Hydrodynamic diameters (D_h) of streptavidin-MPs particle and their conjugates with DNA were measured using Zetasizer Nano ZSP (Malvern Panalytical, Malvern, UK), which features a 4 mW He–Ne laser (633 nm). The 2 µL of the suspensions were diluted in 200 µL of NEB2.1 buffer (NEB) and inserted into a disposable solvent resistant micro cuvette (ZEN0040; Malvern Panalytical, Malvern, UK). Experimental measurements based on noninvasive backscatter technology were taken with a fixed 173° scattering angle at 25 °C. The experiments were initiated after the sample reached thermal equilibrium. Each sample was measured at least four times, and each measurement consisted of 50 acquisitions. The data were collected and analyzed with Zetasizer Software ver. 8.00. The mean of the D_h of different streptavidin-MPs and conjugates were statistically validated by a one-way ANOVA test. The statistic procedures were performed by OriginProLab 11 software (OriginLab Corporation, Northampton, MA, USA).

Section S6. Optimization of the conjugation with streptavidin-MPs and DNA-targets

6.1. Methods

Method of conjugation of labeled DNA with streptavidin-MPs

The streptavidin-MP suspensions (1% w/v, 1-5 µL) were diluted in 30 µL of NEB2.1 buffer, then streptavidin-MPs were separated by a magnetic holder (Evrogen, Moscow, Russia) for removing the storage buffer components. The MPs pellet was incubated with shaking (45-50 round/min) with 30 µL of FAM/biotin-labeled target DNA in different concentrations (25-200 nM for trans-targets and 20 nM or 1 nM for cis-targets) in NEB2.1 buffer for 60 or 10 min (optimal time) at 37°C. The DNA – streptavidin-MP conjugates and unbound DNA were separated using the magnetic holder, supernatants with unbound DNA were collected. The DNA – streptavidin-MP conjugates pellet was washed using NEB2.1 three times and then used for cleavage. The 30 µL of the samples (the initial target DNA solutions (*total*), supernatants with unbound DNA (*supernatant*), and DNA – streptavidin-MP conjugates resuspended in NEB2.1 buffer) were mixed with 70 µL of 25 mM Tris-HCl, pH 9.0 with 50 mM NaCl (F-buffer), after that, the intensity of FAM fluorescence (I) was measured using a black 96-well microplate Fluoro Nunc (Thermo Scientific, Waltham, MA, USA) by EnSpire multimode plate reader (PerkinElmer, Waltham, MA, USA) at 498 nm of excitation wavelength and 517 nm of emission wavelength. Number of flashes was 1000 for samples with 100 nM initial DNA and 3000 flashes for samples with 20 nM initial DNA. Obtained signals for both cases were in linear range of the reader (within 1 million relative fluorescence units). The loading (%) of MPs with DNA was calculated as $(I_{total} – I_{supernatant})/I_{total}$.

6.2. Results

Optimization of the conjugation time

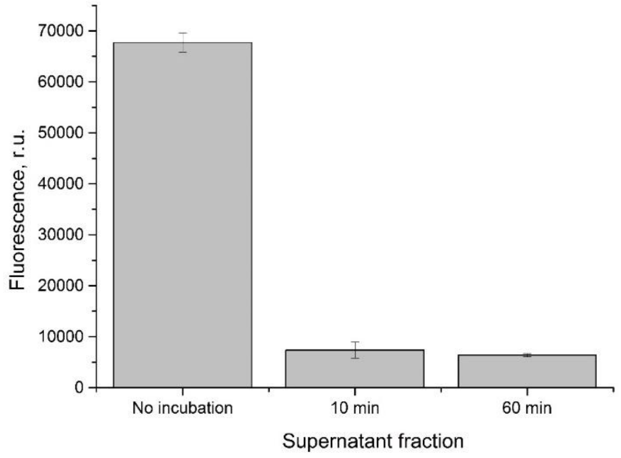

Figure S5. Estimation of the conjugation time for streptavidin-AMPs and biotinylated dsDNA (eGFP-20). Fluorescence was measured in liquid fraction after magnetic separation for 10 min and 60 min incubation of streptavidin-AMPs (1%, 2 µL) and eGFP-20 (100 nM, 30 µL). FAM signal

of unbound eGFP-20 in liquid fraction after magnetic separation was measured and presented as “No incubation” column. R.u. – relative units.

Table S6 Fluorescence quenching of FAM-labeled DNA trans-targets with different lengths bound to MPs.

Length of ds-adaptor, bp	Fluorescence quenching, % ± – standard deviation	
	Symmetric MPs	Asymmetric MPs
0	65.2±2.3	46.3±4.0
20	57.5±3.6	32.3±3.2
40	45.1±1.2	29.3±6.6
80	37.3±1.6	30.3±7.5
120	35.3±3.8	32.8±1.9
160	37.1±5.0	27.5±9.2
300	44.4±10.8	26.9±14.0
500	30.9±6.0	20.3±14.0
1000	27.4±6.8	12.7±10.7

The fluorescence quenching (%) was calculated according to the equation:

$$\text{Fluorescence quenching} = \left(1 - \frac{\text{F (with MP)}}{\text{F (without MP)}}\right) \times 100\%$$

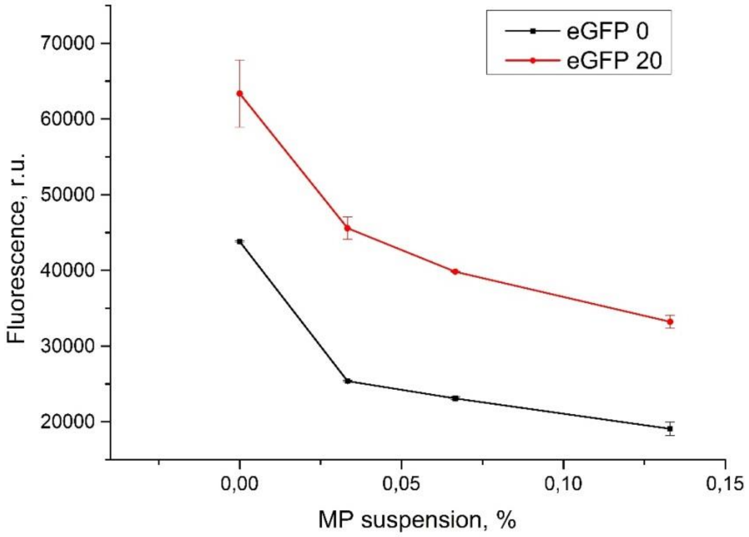

Figure S6. Optimization of the amount of MPs for conjugation with biotinylated dsDNA (eGFP-0 and eGFP-20). R.u. – relative units.

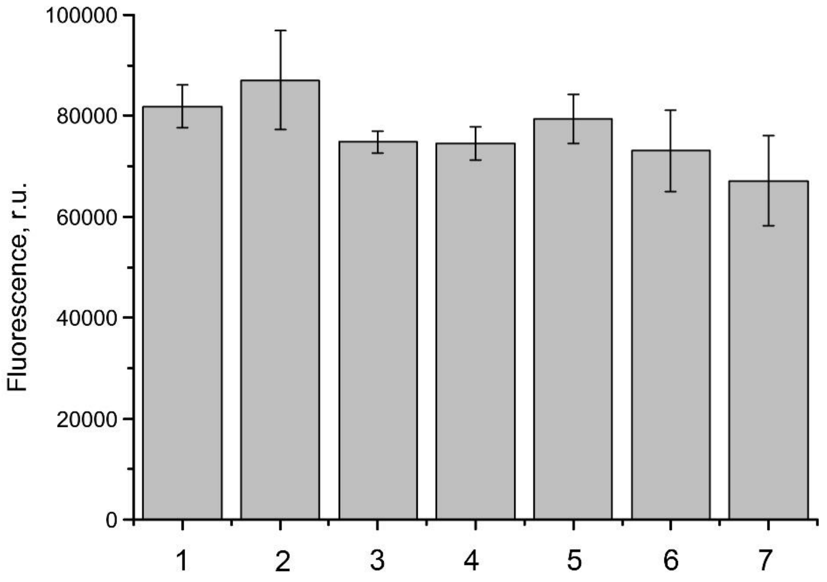

Figure S7. Fluorescence of 100 nM FAM dye in presence of dsDNA, ssDNA (18 nt oligo dT) or asymmetric MPs: 1) 100 nM FAM dye without another components, 2) with 10 µM dsDNA, 3) with 1 µM dsDNA, 4) with 0.1 µM dsDNA, 5) with MP-streptavidin, 6) with MPs-streptavidin + 10 µM dsDNA, 7) MPs-streptavidin + 10 µM ssDNA. R.u. – relative units.

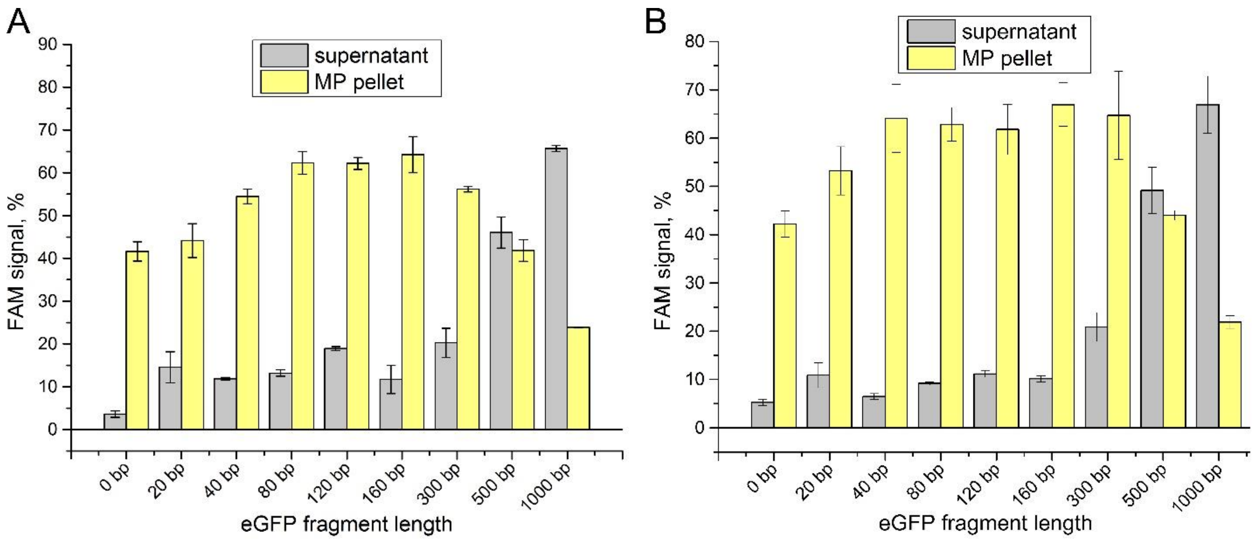

Figure S8. Fluorescence of DNA trans-targets bound (yellow columns) and unbound (grey columns) with MPs after conjugation and magnetic separation. A) Symmetric MPs-streptavidin-SMPs. B) Asymmetric MPs – streptavidin. The initial concentration of each FAM-labeled DNA trans-target was 100 nM.

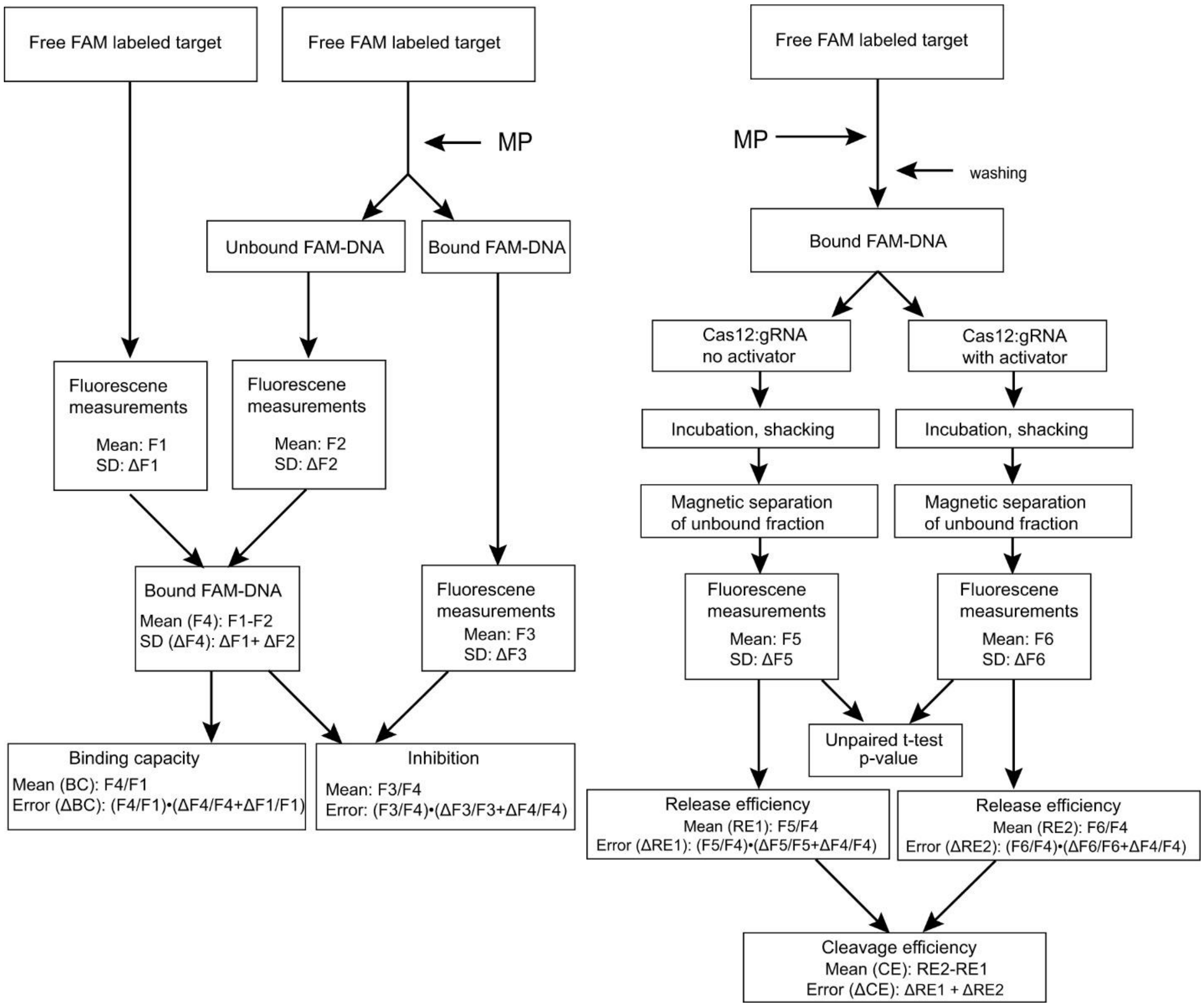

Figure S9. Scheme of experiments and calculations of binding capacity, inhibition (fluorescence quenching), release efficiency, and cleavage efficiency for trans- and cis- targets.

Table S7. Loading of the symmetric and asymmetric MPs-streptavidin with 100 nM biotinylated trans-targets with different length of ds-eGFP-adaptor

Length of ds-adaptor, bp	Trans-target loading, % ± – standard deviation	
	Symmetric MPs	Asymmetric MPs
0	96.4±0.7	94.7±0.6
20	85.4±3.6	89.1±2.6
40	88.1±0.3	93.5±0.6
80	86.8±0.7	90.8±0.3
120	81.1±0.4	88.8±0.7
160	88.3±3.3	89.9±0.64
300	79.8±3.5	80.9±1.49
500	54±3.6	50.8±4.8
1000	34.3±0.7	33.1±6.0

Table S8. Loading of symmetric and asymmetric MPs-streptavidin with biotinylated cis-targets of different length

Length of IGS(I)-cis-targets, bp		Cis-target loading, % ± – standard deviation			
		Symmetric MPs		Asymmetric MPs	
ds-adaptor	total	100 nM cis-target	20 nM cis-target	100 nM cis-target	20 nM cis-target
3	98	87.0±2.4	85.5±0.6	93.5±1.0	85.7±1.7
26	121	89.7±1.4	86.1±1.8	91.2±1.7	83.7±1.8
76	171	91.3±0.6	88.8±1.1	96.2±1.2	89.0±2.0
126	221	91.5±1.2	ND*	95.9±1.3	ND
178	273	89.6±0.3	86.8±0.2	94.4±0.3	87.3±1.6
278	373	88.3±1.9	85.3±0.8	85.8±0.5	86.1±1.7
478	573	81.1±2.0	81.9±0.38	61.9±11.9	84.3±1.3

*ND – not determined

Section S7. Trans-cleavage by Cas12a

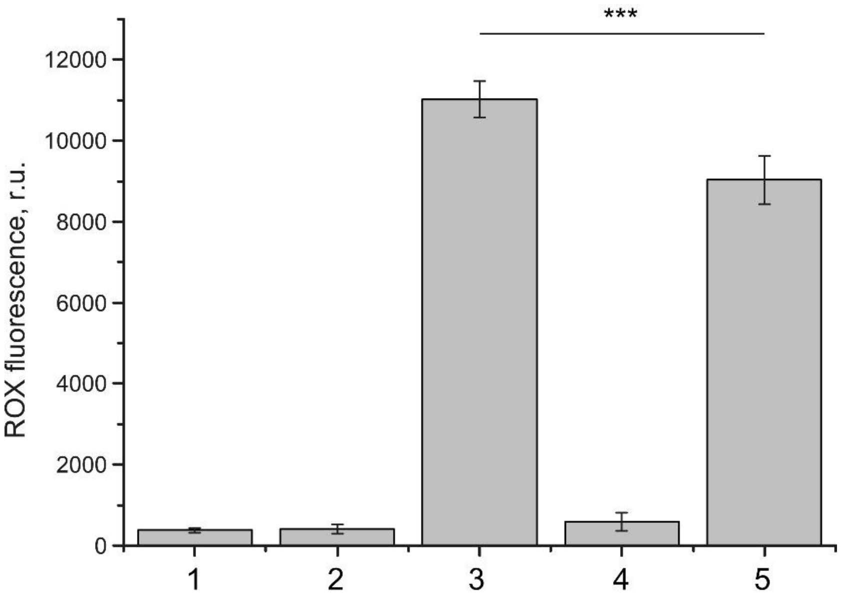

Figure S10. Control measurements of 100 nM ROX probes: 1) ROX-dT15-BHQ2 probe, 2) ROX-dT15-BHQ2 probe in presence of SMPs, 3) ROX-dT15, 4) ROX-dT15-BHQ2 probe in presence of Cas12a without IGS target, 5) ROX-dT15-BHQ2 probe in presence of 66 nM Cas12a-gRNA1 and 3.3 nM IGS target. *** - p-value <0.01. r.u. – relative units.

Before trans-cleavage of DNA – streptavidin-MP conjugates, we verified ROX-dT15-BHQ2 probe as appropriate internal control for Cas12 activity estimation. The probe demonstrated almost completely cleavage under standard condition of the experiment without MPs in reaction mix (**Figure S10**). Non-cleaved probe demonstrated low fluorescence that was less than 10% of signal of ROX-dT15 without the quencher. Probably, the signal was caused incomplete quenching. Thus, the fluorescence of non-cleaved probe did not influence on the detected signal.

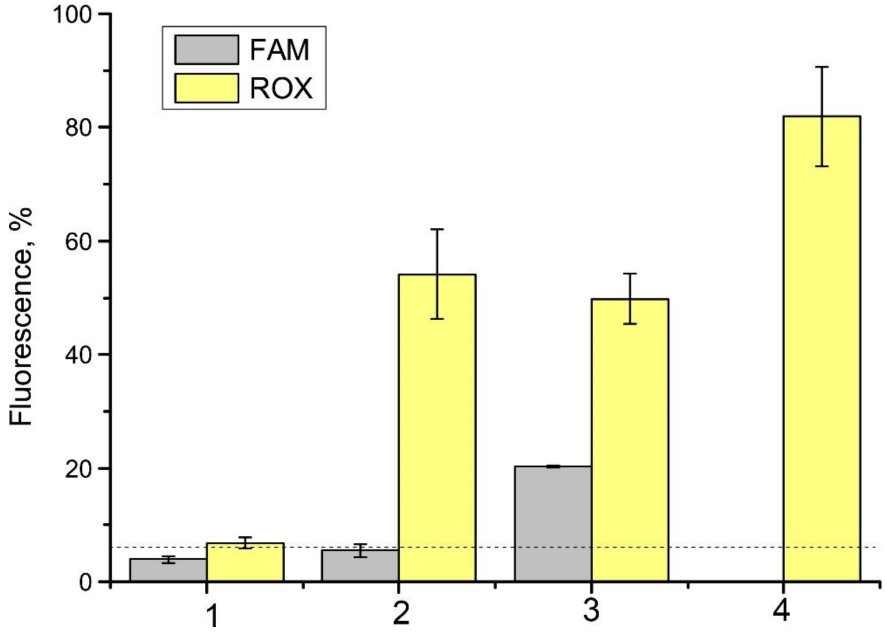

Figure S11. Control of ssDNA fragment (dT15) cleavage in presence of 66 nM Cas12-gRNA1. Florescence of released FAM after cleavage in liquid fraction was normalized to calculated bound FAM target. Fluorescence of ROX in liquid fraction corresponded efficiency of trans-cleavage of Cas12-gRNA1 without impact of target's conjugation to MP. ROX fluorescence after probe cleavage was normalized to fluorescence of ROX-dT15 oligonucleotide of equal concentration (100 nM). 1. ds-eGFP-160 without ssDNA-dT15 conjugated to symmetric MPs after reaction in absence of IGS target. 2) ds-eGFP160 without ssDNA-dT15 conjugated to symmetric MPs after reaction in presence of 3 nM IGS target. 3) eGFP-160 comprised ssDNA-dT15 conjugated to symmetric MPs after reaction in presence of 3 nM IGS target. 4) ROX-dT15-BHQ2 probe after reaction in presence 3 nM IGS, no MPs were added. Dash line indicates background value of fluorescence of ROX-dT15-BHQ2 probe normalized to total ROX signal with added 3 value of SD (three-sigma rule).

We performed control of dsDNA cleavage activity of Cas12. We compared two constructs (without and with ss dT15) based on eGFP-160 conjugated to symmetric MPs. FAM release was no observed in case of eGFP-160 without ssDNA-dT15 (**Figure S11**). The eGFP fragment with ssDNA-dT15 portion display FAM release upon Cas12-sgRNA1 treatment. The experiment showed that Cas12-sgRNA1 did not cleave dsDNA under conditions with MPs. So, all cleavage events of DNA – streptavidin-MP conjugates should be a result of ssDNA-dT15 terminal part. ROX probe cleavage was found to undergo less effective cleavage in presence of MPs (**Figure S11**). ROX probe cleavage in presence of MP-eGFP-160-C3-FAM that unable to be cleaved was the same to those conjugates with cleaved target eGFP-160-dT15-FAM. This result showed no competition between MP-conjugated target and free target. Thus, the less ROX cleavage could indicate the inhibitor of Cas12 activity by MPs.

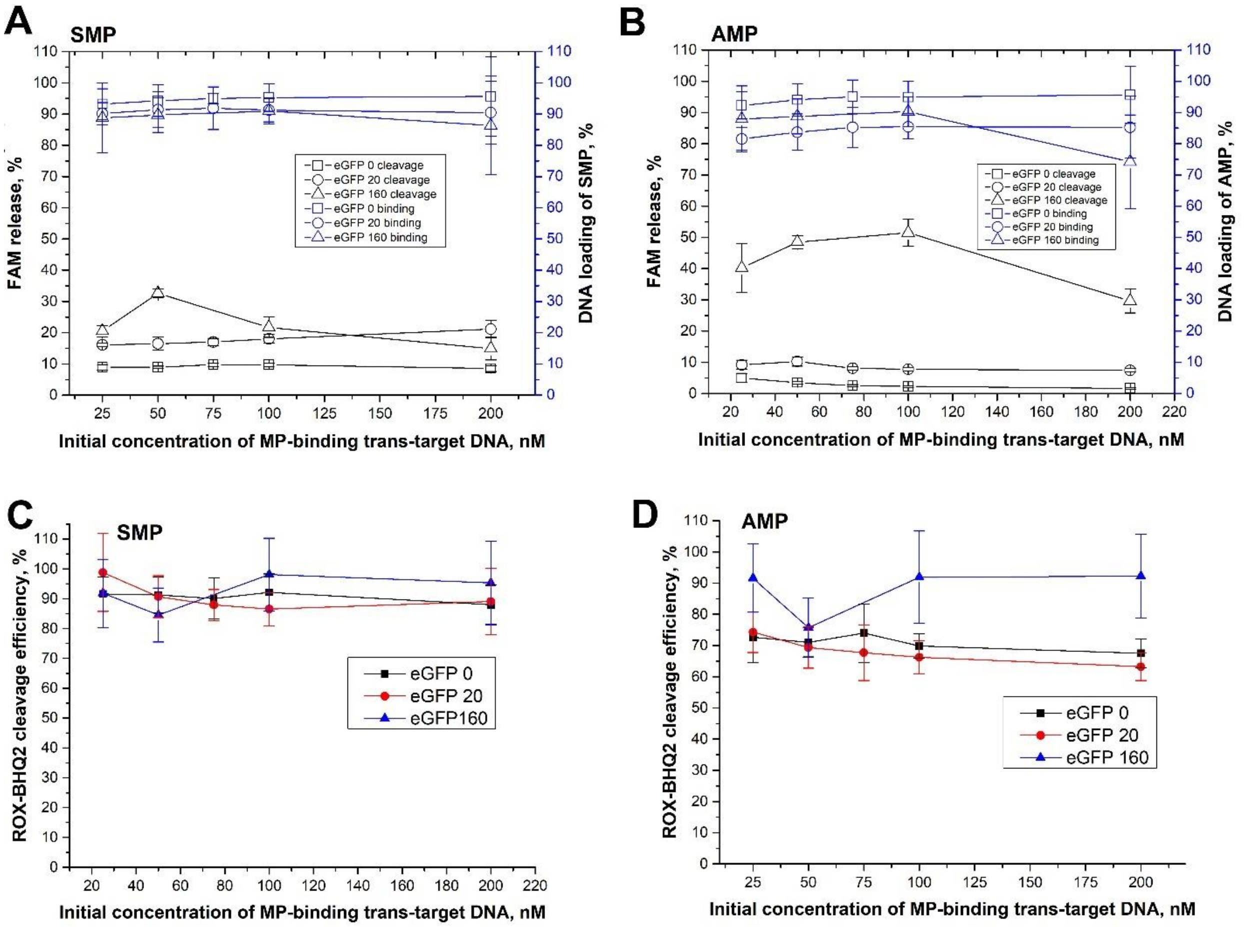

Figure S12. Cleavage of trans-target with eGFP-0, eGFP-20, eGFP-160 upon different concentration of the targets and constant concentration of MPs (0.1%). A) Results for symmetric MP – trans-target conjugates, B) Results for asymmetric MP – trans-target - conjugates, C) Control cleavage of ROX-dT15-BHQ2 (100 nM) with symmetric MP-– trans-target conjugates, D) Control cleavage of ROX-dT15-BHQ2 (100 nM) with asymmetric MP – trans-target conjugates

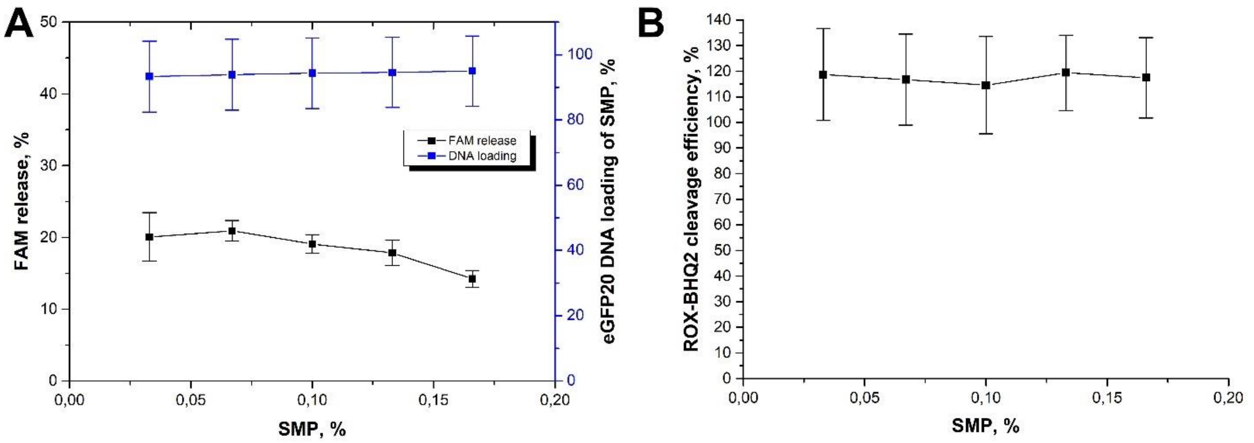

Figure S13. Trans-cleavage of 100 nM trans-target with eGFP-20 conjugated with SMPs of different concentrations. A) FAM signal of symmetric MP– trans-target conjugates, B) Control cleavage of ROX-dT15-BHQ2 (100 nM) with symmetric MP– trans-target conjugates.

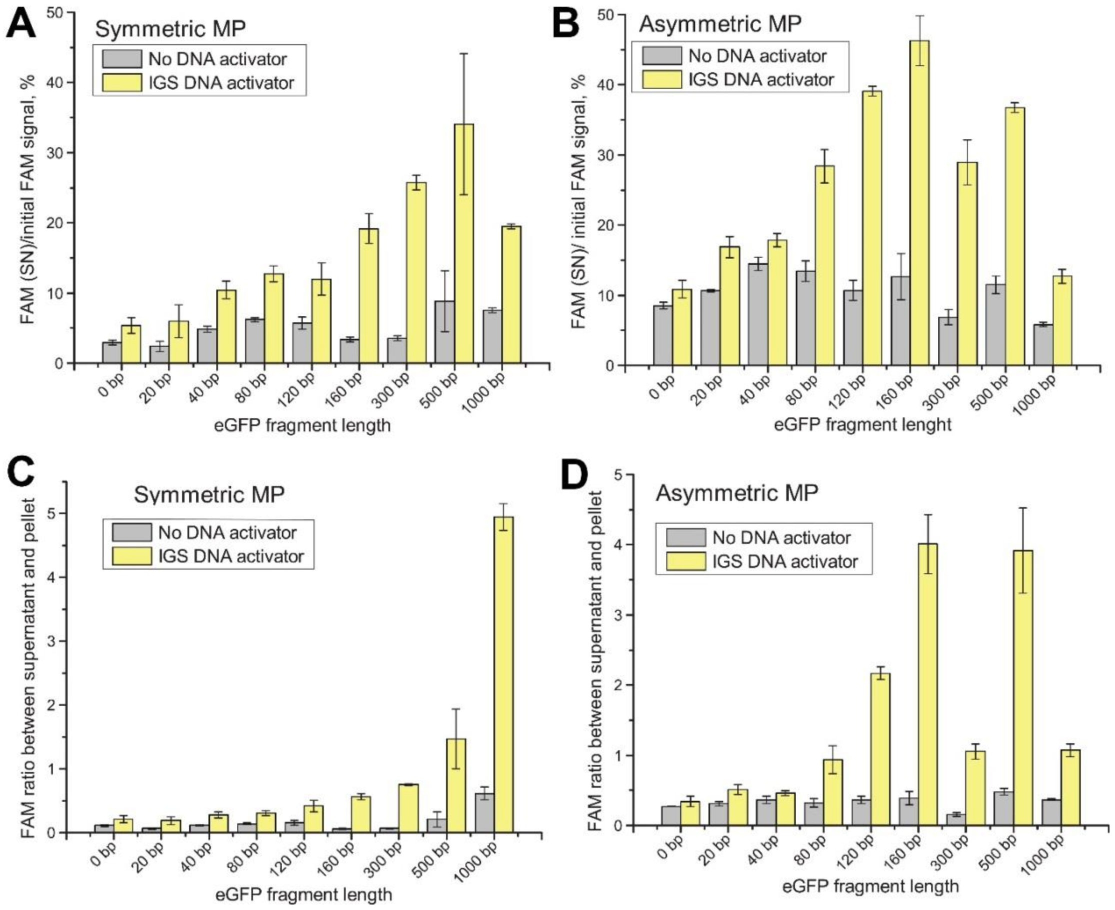

Figure S14. Interpretation of experiment for trans-cleavage efficiency assay. Ratio of FAM signal in supernatant after the reaction with symmetric (A) or asymmetric (B) MP-conjugates to initial signal of FAM-labeled eGFP fragment before conjugation. No correction for capacity MP to eGFP fragment was considered. Distribution of FAM signal between supernatant and symmetric (C) or asymmetric (D) MP pellet after reaction. SN – supernatant.

Section S8. Cleavage of DNA conjugated with MPs using activate-independent endonuclease (DNAseI)

8.1. Methods

The pellets of trans-targets DNA – streptavidin-MP conjugates (100 nM trans-targets DNA, 2 µL of 1% MP suspension) were diluted in 30 µL –10 mM Tris-HCl pH 7.6, $MgCl_2$ 2.5 mM, $CaCl_2$ 0.5 mM (DNAseI buffer) instead of NEB2.1 buffer. Then 3 µL of DNAseI was added to the conjugate and incubated at 37°C for 30 min. The reaction was stopped by the addition of 25 mM EDTA. The fluorescence of FAM for the bound and cleaved probe was detected as described in Section 2.7. The fluorescence of FAM in the MP pellet (bound probe) and the supernatant (cleaved probe) separated by the magnet was estimated as described in Section "Trans-cleavage of DNA attached to MPs by Cas12a". The DNaseI inactivated at 75°C for 15 min was used as control of background FAM release.

8.2. Results

The shortest target with eGFP-0 had significantly less efficiency for DNAseI-mediated degradation. The targets with eGFP-20 – eGFP-500 conjugated to symmetric MPs showed close efficiency upon the cleavage. The conjugates of asymmetric MPs with eGFP-20 – eGFP-500 demonstrated maximal efficiency within eGFP length of 20 – 80 bp. The longest eGFP-1000 demonstrated surpass efficiency for cleavage, but with a low ratio of specific and non-specific release values. This high non-specific release (also reported for Cas12a experiments, see section 2.3) could be caused by the dissociation of eGFP-1000 from MPs or partial MP degradation. In contradistinction to Cas12a, DNAseI was able to degrade the conjugates with all lengths of ds-ss DNA targets. As well as even one cleavage event was sufficient for FAM release, the remaining ds-ssDNA molecule available for cleavage becomes an overabundant substrate and therefore, reduces the beneficial effect influencing the FAM release. Probably this by-side process caused the decrease of cleavage efficiency for longer eGFPs. The average % of FAM release was ~50-60% for both eGFP-20 – eGFP-500 (symmetric MP) and eGFP-20 – eGFP-80 (asymmetric MP).

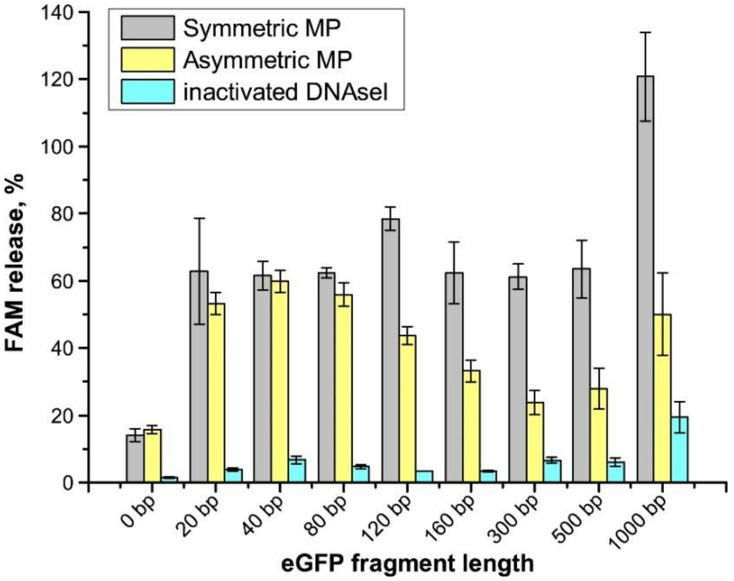

Figure S15. Dependence of DNAseI cleavage efficiency on ds-adaptor length for trans-targets conjugated to different MPs.

Section S9. Detection of cis-cleavage of DNA conjugated with MPs by gel-electrophoresis

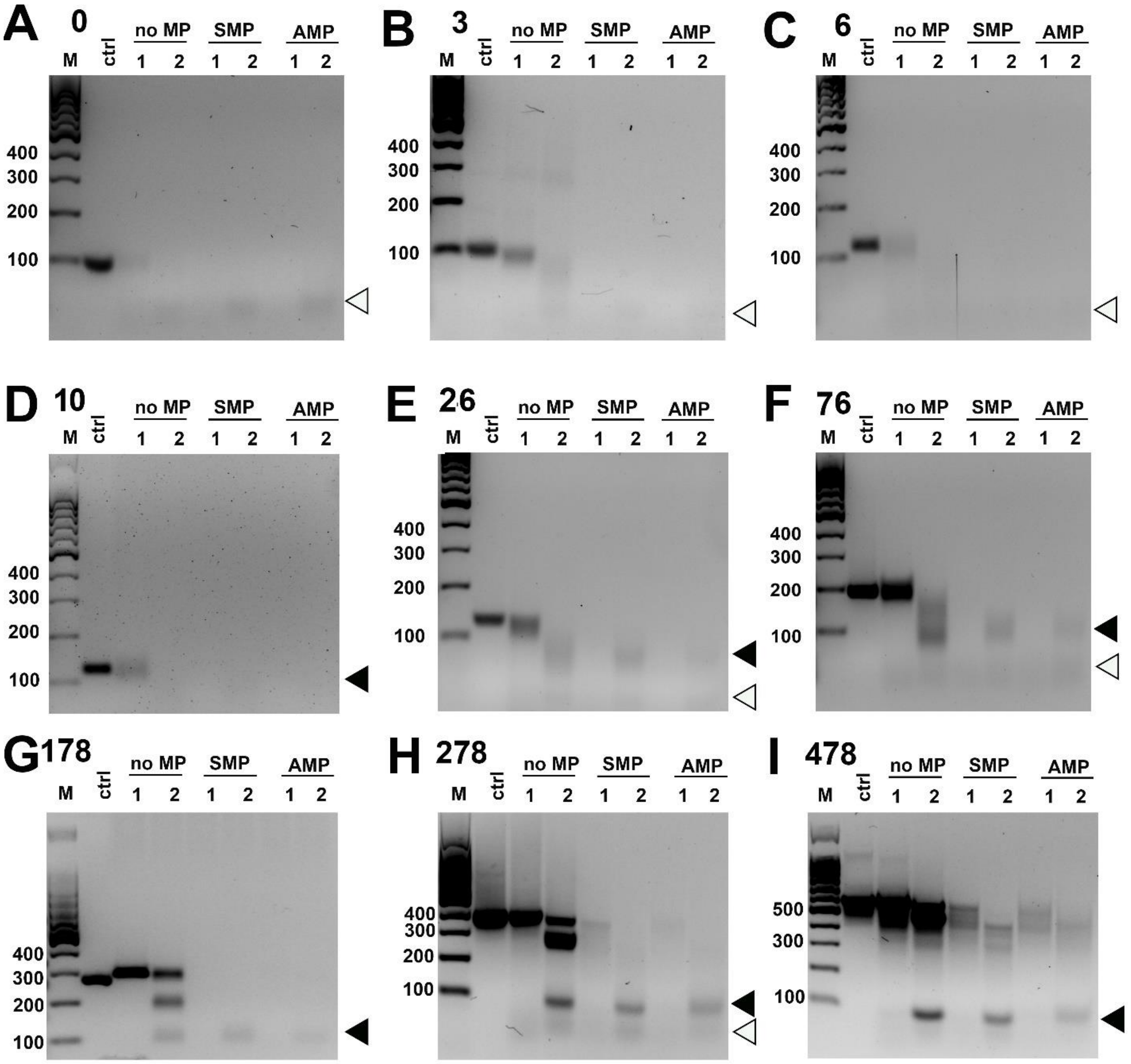

Figure S16. Cis-cleavage of DNA (IGS(I)) – streptavidin-MP conjugates differing the ds-adaptor length between spacer (20 bp) and MP surface. Scans of gels after electrophoresis of liquid fraction after Cas12 cleavage of conjugates with different ds-adaptor lengths: 0 – 478 bp. M – dsDNA ladder, ctrl – non-cleaved cis-target with the corresponding length, no MP – cis-cleavage in solution, 1 – gRNA1 (non-cognate), 2 – gRNA2 (cognate). Black triangle represents a 97 bp fragment of cis-cleavage, while white triangle represents the gRNA.

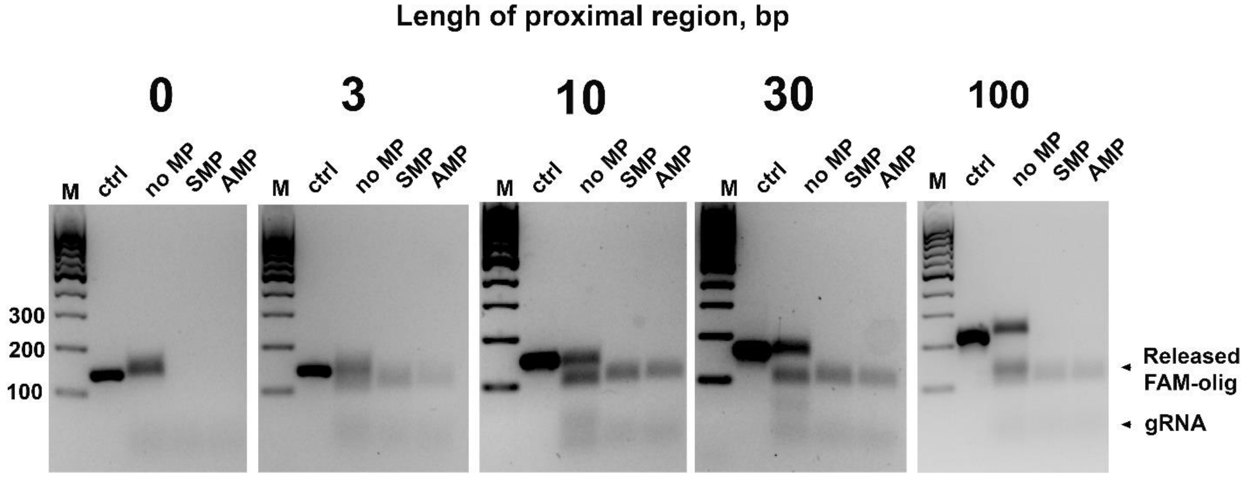

Figure S17. Cis-cleavage of DNA (IGS(II)) – streptavidin-MP conjugates differing the ds-adaptor length between PAM and MP surface. Scans of gel after electrophoresis of liquid fraction after Cas12a cleavage of conjugates with different ds-adaptor lengths: 0- 100 bp. M – DNA ladder, ctrl – activator IGS DNA before conjugation. Released FAM-olig represents 126 bp FAM-labeled IGS product of cis-cleavage**.**

Section S10. Visualization of Cas12-gRNA -DNA complex

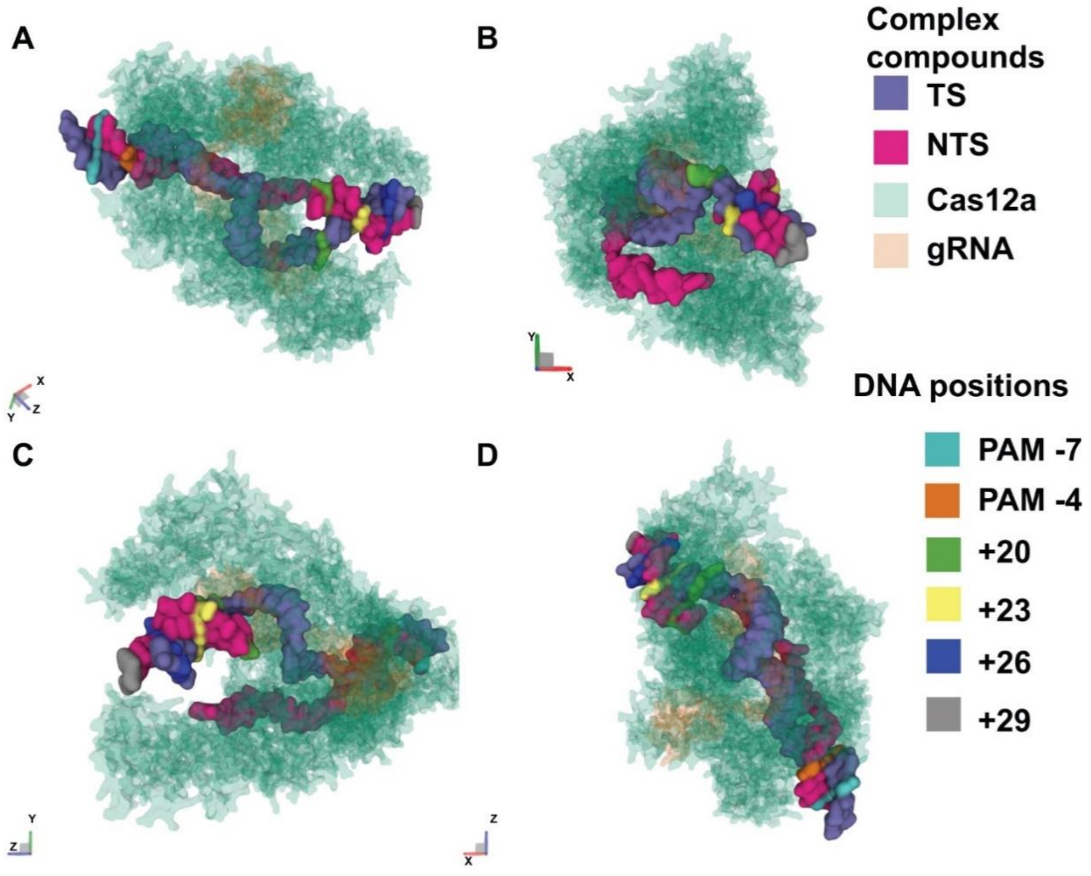

Figure S18. Visualization of the complex of catalytically inactive FnCas12a, crRNA guide and dsDNA target based on PDB 6I1K: chosen view (A) and three projections for the PDB view – XY (B), YZ (C) and XZ (D).

PDB data for LbCas12a are limited to the structures of the protein itself and the protein-gRNA complex. Thus, the use of a protein ortholog (FnCas12a), which represented the most complete structure of the Cas12-gRNA-DNA complex, was the most effective tool for interpreting the experimental results. The comparative approach based on the use of Cas12a orthologs for the evaluation of Cas12-gRNA-DNA complexes was successfully applied in particular by Cofsky et al.[39] The visualization showed that the DNA region of the corresponding R-loop at +20 - +26 nt positions was in a pocket of Cas12 that caused steric obstacles (Figure S16A, Supporting Information). We proposed that in the cases of short (+23 and +26) IGS DNA, streptavidin interacting with the biotinylated tail of cis-target was near the DNA-Cas12 at the Cas12a side where the enzyme lobes (RuvC) form clefts (Figure S16, Supporting Information). Feasibly, the close location of streptavidin or MP-streptavidin to the RuvC impacted the Cas12a functioning. The transition from short (+23 and +26) to longer (+27 and +29) DNA accords to the movement of the binding site with streptavidin beyond the enzyme lobes (Figure S16A, C, Supporting Information), and the Cas12 functioning was facilitated.

Section S11. Cis-cleavage of DNA conjugated with MPs

10.1. Methods

Estimation of cis-cleavage of DNA attached to MPs via fluorescence of the trans-target

Indirect assessment of cis-cleavage was carried out by registration of the fluorescence of the cleaved FAM-dT15 BHQ1 probe when Cas12a was activated by cis-target DNA attached to MPs. The premix of gRNA-Cas12a (100 µL, 66 nM gRNA1 (or gRNA2), 66 nM EnGene LbCas12a, incubation at 25°C for 10 min) was added to the pellet of cis-target DNA – streptavidin-MP conjugate (10 µL, 1 nM cis-targets DNA, 2 µL of 1% MP suspension) and 500 nM FAM -dT15-BHQ1 probe. The reaction was proceeded at 37°C for 30 min upon shacking. After 5, 10, 20, 30 min, 30 µL of the mix was taken and treated with 50 mM EDTA to stop the reaction, the magnetic holder was applied, and fluorescence of FAM in supernatant and pellet fractions was measured as described in Section "Trans-cleavage of DNA attached to MPs by Cas12a".

10.2. Results

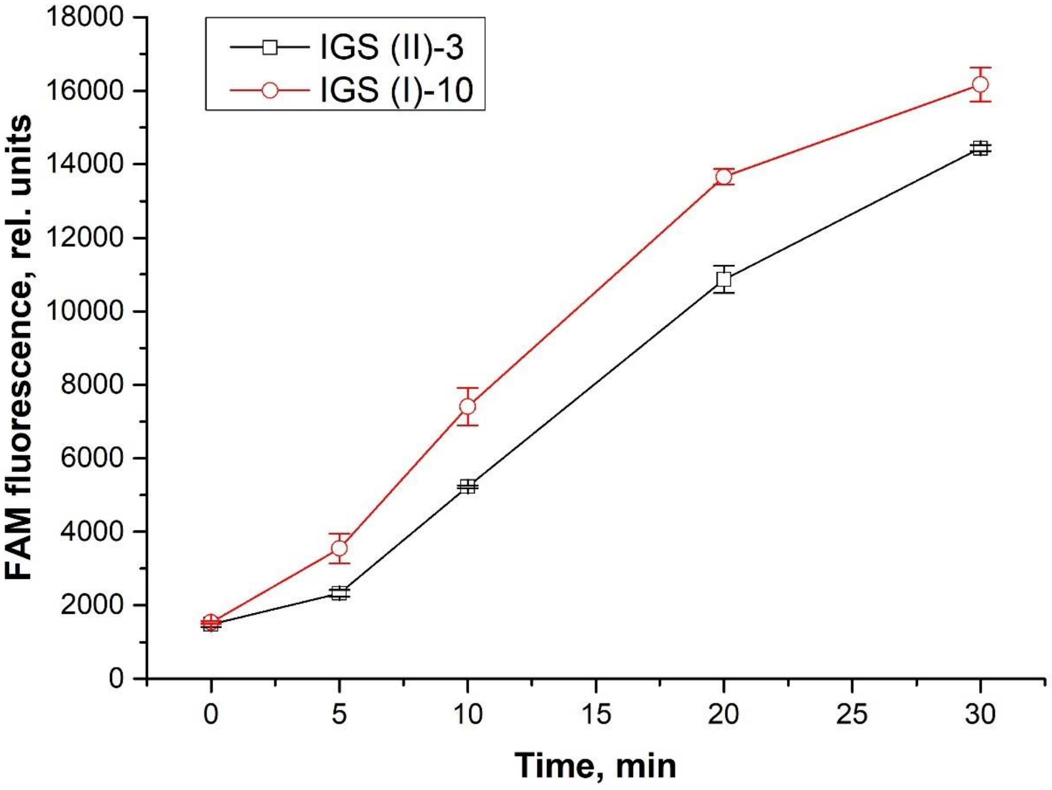

Figure S19. Effect of cis-target orientation on SMP surface on trans-cleavage efficiency of free probe. 1 nM cis-targets were used for conjugation to 0.0625% SMP. The conjugation of IGS(II)-3 positionated PAM closer to SMP surface. The conjugaton of IGS(II)-10 positionated 3' part of spacer closer to SMP surface. Orientation of the cis-targes on the MP surface provided different states of activated Cas12a – linked to SMP surface (IGS(I)-3) or Cas12a released in solution (IGS(II)-10). 100 nM FAM-dT15-BHQ1 probe was used for detection of of trans-cleavage.
